# Evaluating a worldwide wheat collection for resistance to Hessian fly biotype ‘Great Plains’

**DOI:** 10.3389/fpls.2024.1402218

**Published:** 2024-05-22

**Authors:** Yunfeng Xu, Nida Ghori, Shabbir Hussain, Xiaoting Xu, Zhenqi Su, Dadong Zhang, Lanfei Zhao, Xuming Liu, Ming-Shun Chen, Guihua Bai

**Affiliations:** ^1^ Department of Agronomy, Kansas State University, Manhattan, KS, United States; ^2^ Hard Winter Wheat Genetics Research Unit, Agricultural Research Service, US Department of Agriculture (USDA-ARS), Manhattan, KS, United States; ^3^ Agricultural Genomics Institute at Shenzhen (AGIS), Chinese Academy of Agricultural Sciences (CAAS), Shenzhen, China

**Keywords:** Hessian fly, new resistant sources, biotype GP, *Triticum aestivum*, genetic improvement

## Abstract

Hessian fly (HF), *Mayetiola destructor*, is a major insect pest that causes severe losses in grain yield and quality of wheat (*Triticum aestivum*). Growing resistant cultivars is the most cost-effective approach to minimize wheat yield losses caused by HF. In this study, 2,496 wheat accessions were screened for resistance to the HF biotype ‘Great Plains’ (GP) in the greenhouse experiments. To purify seeds from heterogeneous resistant accessions, we recovered single resistant plants from 331 accessions that had at least one resistant plant after HF infestation of a global collection of 1,595 accessions and confirmed 27 accessions with high resistance (HR), and 91 accessions with moderate resistance (MR) to the GP biotype using purified seeds. Screening of 203 U.S. winter wheat accessions in three experiments identified 63 HR and 28 MR accessions; and screening of three additional Asian panels identified 4 HR and 25 MR accessions. Together, this study identified 96 HR accessions and 144 MR accessions. Analysis of the geographic distribution of these HR and MR accessions revealed that these countries with HF as a major wheat pest usually showed higher frequencies of resistant accessions, with the highest frequency of HR (81.3%) and MR (30.6%) accessions identified from the U.S. In addition, phenotyping of 39 wheat accessions that carry known HF resistance genes showed that all the accessions except *H1H2* remain effective against GP biotype. Some of these newly identified resistant accessions may contain new HF resistance genes and can be valuable sources for developing HF resistant wheat cultivars.

## 1 Introduction

Hessian fly (HF, *Mayetiola destructor*) is one of the most destructive pests in wheat (*Triticum aestivum*) and causes significant economic losses in the U.S. and many other countries (Ratcliffe and Hatchett, 1997; Chen et al., 2009; Shukle et al., 2010). Genetic resistance is the most effective and economical strategy for HF control (Berzonsky et al., 2003). To date, a total of 18 HF biotypes, including biotypes A to O, GP (Great Plains), v*H9*, and v*H13*, have been reported based on their differential responses to a set of resistance genes (Formusoh et al., 1996; Ratcliffe and Hatchett, 1997; Zantoko and Shukle, 1997). Biotype GP is the most prevalent HF biotype in the U.S. Great Plains (Sardesai et al., 2005; Chen et al., 2009; Garcés-Carrera et al., 2014; Tan et al., 2017).

The HF resistance genes fit the gene-for-gene model (Hatchett and Gallun, 1970), meaning that a specific HF resistance gene in plants can provide resistance only to HF carrying the corresponding avirulence gene. New biotypes occur due to rapid co-evolution in response to the continuous deployment of existing resistance genes, resulting the loss of resistance of existing resistance genes (Cambron et al., 2010). Therefore, searching for new sources with HF resistance and stacking multiple resistance genes from different sources in new wheat cultivars are essential and effective strategies to improve the durability of wheat cultivars for HF resistance (Shukle et al., 2016).

To date, 37 HF resistance genes (*H1* to *H36* and *Hdic*) have been reported (Bassi et al., 2019; Xu et al., 2021) with 11 (*H1*-*H5*, *H7*, *H8*, *H12*, *H34*, *H35*, *H36*) from common wheat (Ratcliffe and Hatchett, 1997; Williams et al., 2003; Sardesai et al., 2005; Li et al., 2013; Zhao et al., 2020). The others were identified from wheat relatives, including six (*H13*, *H22*, *H23*, *H24*, *H26*, and *H32*) from *Aegilops tauschii*, 15 (*H6*, *H9*-*H11*, *H14*-*H20*, *H28*, *H29*, *H31*, and *H33*) from durum wheat, two (*H21* and *H25*) from *Secale cerale* L. (RR, 2n = 2× =14), and one from *Ae*. *ventricosa* (*H27*), one from *Ae*. *triuncialis* (*H30*), and one from emmer wheat (*Hdic*) (Martin-Sanchez et al., 2003; Kong et al., 2005; Liu et al., 2005; Kong et al., 2008; Bassi et al., 2019; Zhao et al., 2020). In the last several decades, these genes have been important HF resistant sources for wheat genetic improvement. However, Chen et al. (2009) found that only five genes (*H13*, *H21*, *H25*, *H26*, and *Hdic*) were highly resistant to all six HF populations collected from Texas, Oklahoma, and Kansas. Garcés-Carrera et al. (2014) showed that eight genes (*H12*, *H13*, *H17*, *H18*, *H22*, *H25*, *H26*, and *Hdic*) were highly resistant to the five HF populations collected from Texas, Louisiana, and Oklahoma. Other surveys reported that six genes (*H12*, *H18*, *H24*, *H25*, *H26*, and *H33*) are still resistant to HF populations collected from the southeastern U.S., with *H12* and *H18* only partially effective. Some of the resistance genes, such as *H24*, *H25*, and *H26*, are associated with undesirable agronomic traits when introgressed into elite wheat (Cambron et al., 2010; Shukle et al., 2016). Continuous searching for new sources of HF resistance from adapted wheat cultivars or elite breeding lines can not only provide new sources of HF resistance genes, but also facilitate quick release of new cultivars. These sources can streamline the breeding process, allowing for the more efficient integration of resistance traits into existing, well-adapted lines.

In the current study, a total of 2,496 wheat accessions collected worldwide were screened for their responses to HF biotype GP. The objectives of this study were to (1) identify new sources of HF resistance, (2) study the geographic distribution of HF resistant accessions, and (3) purify selected resistant accessions for breeding using single plant reselection.

## 2 Materials and methods

### 2.1 Plant materials

Wheat accessions from several different sources were screened for resistance against HF biotype GP. A global collection of 1,595 accessions was obtained from the United States Department of Agriculture-Agricultural Research Service (USDA-ARS), National Small Grains Collection (NSGC), Aberdeen, Idaho. Due to space limitation, 407 accessions (NSGC407) were screened in fall 2019, and 1,188 accessions (NSGC1188) were screened in spring 2020. In addition, four panels from Iran, Pakistan, East Asia and the U.S. were also screened for HF resistance, including a panel of 369 Iranian varieties and landraces (IRAN369) (Alipour et al., 2017) a panel of 176 Pakistan accessions (PAK176), a panel of 153 East Asia accessions (EA153), and a panel of 203 U.S. winter wheat accessions (AM203). The EA153 panel contains 123 wheat accessions from China, 27 from Japan, and three from South Korea. The U.S. AM203 panel includes 137 hard winter wheat and 66 soft winter wheat accessions, with 19 released cultivars and 184 elite breeding lines from various U.S. winter wheat nurseries (Zhang et al., 2010) and all the 203 accessions were derived from single seeds.

In addition, 39 wheat accessions that carry known HF resistance genes including *H1H2*, *H3*, *h4*, *H5*, *H6*, *H7H8*, *H9*, *H10*, *H11*, *H12*, *H13*, *H14*, *H15*, *H16*, *H17*, *H18*, *H19*, *H20*, *H21*, *H22*, *H23*, *H24*, *H25*, *H26*, *H28*, *H29*, *H31*, *H32*, *H33*, *H34*, *H35H36*, and *Hdic* were evaluated for resistance against biotype GP. ‘Carol’ with *H3*, ‘Caldwell’ with *H6*, and ‘Molly’ with *H13* were used as resistant controls, and ‘Danby’ was used as the susceptible control in all greenhouse experiments.

### 2.2 HF resistance evaluation and resistant line purification

Wheat accessions were screened for HF resistance in the greenhouse at Kansas State University using the same protocol described previously (Xu et al., 2021). In brief, the growth medium was a mixture of soil, sand, and vermiculite (2:1:1). Each planting tray had 12 full rows, which were further divided into 24 half-rows for planting 24 accessions with approximately 20 seeds per accession. The four control cultivars were planted in the four middle half-rows. The greenhouse temperature was set at 18°C with a 14/10 h (light/dark) photoperiod.

At the 1.5-leaf stage, HF biotype GP adults were released onto wheat seedlings in the trays covered by a cheesecloth tent for infestation. The tent was used to maintain high humidity and restrict movement of the flies inside the tent and was then removed after about one week when larvae hatched from eggs. HF damage on infested seedlings was scored about three weeks after infestation. Resistant plants usually grow normally with light green leaves and dead larvae at the bottom of the leaf sheath, although some tiny larvae could survive on some resistant plants from time to time. Susceptible plants were stunted with dark green leaves and with live larvae between leaf sheaths at the base. The percentage of resistant plants per line was calculated as the HF resistance score for each accession, which was rechecked one week after the first rating to ensure data accuracy. The accessions with HF resistance scores >50% were designated highly resistant (HR), the accessions with HF resistance scores 1–50% were designated moderately resistant (MR), while the accessions with HF resistance scores 0 were designated susceptible (S).

Since most of the accessions screened in this study have not been selected for HF resistance during the breeding process, it is expected that many accessions that were identified as resistant accessions in this study remain heterogeneous with mixed resistant and susceptible genotypes. Therefore, these accessions with some resistant plants were purified by recovering one resistant plant per line from the HF phenotyping experiments and then their progeny were phenotyped again to confirm their HF resistance. The progeny seeds derived from the single plant were maintained as the seed stock for the resistant accession after phenotypic confirmation. However, all the accessions in the U.S. AM203 panel were derived from single seed selection, and heterogeneity is not expected in the panel; therefore, only these lines with HF resistance observed in at least two experiments were designated as resistant and other accessions with a low level of HF resistance observed in only one experiment were considered as susceptible genotypes.

### 2.3 Statistical analysis

The correlation of HF resistance scores of the U.S. AM203 panel among experiments was calculated using the CORREL function in Microsoft Excel 365 for Windows 10 (Microsoft Corporation, Redmond, WA).

## 3 Results

### 3.1 HF resistant accessions in the worldwide wheat collection

**Figure 1:**
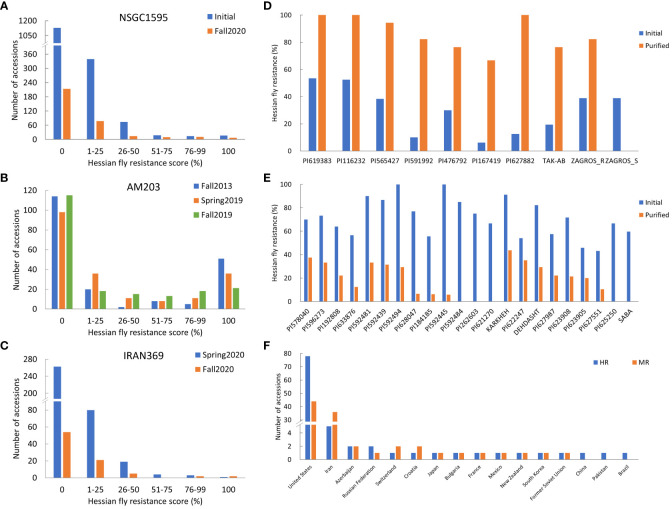
Frequency distribution of Hessian fly resistance scores (%) for the three panels: worldwide panel NSGC1595 **(A)** combining the two subpanels NSGC407 and NSGC1188, the U.S. panel AM203 **(B)**, and the Iranian panel IRAN369 **(C)**; the Hessian fly resistance scores (%) of selected resistant lines that showed significantly improved resistance scores **(D)** and reduced resistance scores **(E)** in the fall 2020 experiment (after purification) than in fall 2019 and spring 2020 experiments (initial screening); and the number of accessions with highly- and moderately-resistant (HR and MR) to HF biotype GP for the countries contributing at least one HR accession **(F)**. In **(A, C)**, the selected accessions with resistant plants were further purified from a single plant per line and screened in fall 2020.

A total of 1,595 worldwide accessions from the NSGC were screened in two separate experiments (**Figure 1A**). Among them, 461 accessions had at least one resistant plant per line, whereas the other 1,134 accessions were completely susceptible (**Figure 1A**). The resistant accessions include 48 HR accessions with the resistance score of 51–100%, and 413 MR accessions with the scores of 1–50% (**Figure 1A**). Among the 48 HR accessions, 16 accessions showed complete resistance with a resistance score of 100%.

To purify the 461 resistant accessions that had at least one resistant plant, one survived seedling per accession was recovered from the HF infested seedling trays for seed increase and purification. Due to HF damage, only 331 accessions produced seeds and were further screened for HF resistance in fall 2020. One hundred and eighteen (35.6%) of them showed various levels of HF resistance, including 27 HR and 91 MR (**Figure 1A**, **Table 1**; **Supplementary Table S1**). The remaining 213 (64.4%) lines were completely susceptible (**Figure 1A**). Among the 27 HR accessions, six (PI 613323, PI 512337, PI 469271, PI 562382, PI 619383, and PI 116232) showed complete (100%) HF resistance, and 21 showed high levels (51–99%) of HF resistance (**Table 1**). Six accessions (PI 619383, PI 116232, PI 565427, PI 591992, PI 476792, and PI 167419) exhibited significant improvement of their HF resistance scores after seed purification (**Figure 1D**, **Table 1**). However, 20 HR lines that had resistance scores > 55.6% in the first round of screening showed a slight lower HF resistance scores < 41.2% and some even completely lost HF resistance in the second screening using the purified seeds from a single plant (**Figure 1E**; **Supplementary Table S1**).

**Table 1:** The newly identified highly resistant accessions with Hessian fly resistance scores > 50% from the global collection (NSGC1595) and four other panels from Iran (IRAN369), Pakistan (PAK176), East Asia (EA153) and the U.S. (AM203).

Accession name (ID)	Country/States^a^	HF score (%)^b^	Accession Name	State	HF score (%)
NSGC1595			AM203		
Lathrop (CItr13457)	United States	78.6	OK05903C	OK	51.7
Solid Straw Tuscan (PI116232)	New Zealand	100	KS970093-8-9-#1	KS	57.4
CIGM90.1291 (PI613323)	Mexico	100	CO03W239	CO	53.3
99ID388 (PI619354)	United States	80	SD05118	SD	88.2
99ID389 (PI619355)	United States	76.5	NE02558	NE	90
NWX008075 (PI619379)	United States	75	KS05HW121-2	KS	96.5
NWX008109 (PI619383)	United States	100	KS970187-1-10	KS	83.3
Nebr. Sel. 521632 (CItr13019)	United States	95	SD05210	SD	54.4
Downy (CItr17421)	United States	95	TX04M410164	TX	92.2
OH 106 (CItr17561)	United States	53.8	Chisholm (PI 486219)	OK	80
Kirda Boogda (PI73361)	Azerbaijan	92.9	NE06619	NE	100
1650 (PI73362)	Azerbaijan	53.3	OH02-7217	OH	100
Nord Desprez (PI167419)	France	66.7	KS020304K~3	KS	86.7
Bojka (PI323408)	Croatia	60	TX06A001376	TX	74.1
Rothenbrunnen 32 (PI351172)	Switzerland	64.7	OH03-41-45	OH	93.2
SO-6300 (PI476792)	Former Soviet Union	76.5	NE06436	NE	86.7
5V254krlVr90 (PI564353)	Bulgaria	88.2	TX06A001431	TX	100
Lutescens 321 (PI565427)	Russian	94.4	OK05511	OK	53.3
Yugtina (PI591992)	Russian	82.4	SD07204	SD	100
HBF0290-146 (PI592441)	United States	61.1	KY96C-0769-7-3	KY	100
IWA8602602 (PI627521)	Iran	63.2	P03207A1-7	IN	100
N02Y4648 (PI633777)	United States	68.8	Roane	VA	100
P921696 (PI633876)	United States	93.3	SD05W148-1	SD	71.7
Clark (PI512337)	United States	100	KS010514-9TM-10	KS	100
Overland (PI647959)	United States	81	N02Y5117	NE	58.3
Wheaton (PI469271)	United States	100	INW0411	IN	100
Freedom (PI562382)	United States	100	Branson	IN	100
**IRAN369**			IL00-8530	IL	98.3
IWA8602448 (PI627466)	Iran	100	IL02-18228	IL	100
IWA8604097 (PI627882)	Iran	100	KS07HW117	KS	100
ZAGROS	Iran	82.4	NE06549	NE	80
TAK-AB	Iran	76.5	IL02-19463	IL	100
**PAK176**			Mocha exp.	OH	93.3
PARVAZ-94	Paksitan	100	Pioneer Brand 26R61	IN	55.9
**EA153**			M03-3616-C	IN	100
Chokwang	South Korea	100	W98007V1	SC	100
**AM203**			Arena exp.	OH	90
KS05HW136-3	KS	74.5	W98008J1	SC	98.3
NI04420	NE	88.8	OK06210	OK	94.8
Duster	OK	100	G69202	IN	66.7
NE04424	NE	91.7	OK06345	OK	75
SD06165	SD	91	D04-5012	AR	100
HV9W03-539R	KS	70.6	G59160	IN	56.7
SD06173	SD	100	GA991336-6E9	GA	58.2
NE05548	NE	91.7	OK05134	OK	62.2
Trego	KS	53.3	OK06848W	OK	100
HV9W03-696R-1	KS	100	GA991371-6E13	GA	65
SD03164-1	SD	73.3	MO040152	MO	86.3
NW04Y2188	NE	96.7	P04287A1-10	IN	100
NE05549	NE	76.7	M04-4715	IN	100

a In the AM203 U.S. panel, the abbreviations for states where the accessions were developed include Arkansas (AR), Colorado (CO), Kansas (KS), Kentucky (KY), Illinois (IL); Indiana (IN), Missouri (MO), Nebraska (NE), Oklahoma (OK), Ohio (OH), South Carolina (SC), South Dakota (SD), Texas (TX), and Virginia (VA).

b The Hessian fly resistance scores of purified seeds was listed for each accession in the NSGC1595, IRAN369, PAK176 and EA153 panels. The mean value was listed for each accession in the U.S. AM203 panel.

### 3.2 HF resistance in the U.S. winter wheat

The AM203 panel consists of 203 winter wheat elite breeding lines and cultivars from the U.S. (**Supplementary Table S2**). In the panel, 91 accessions showed various levels of HF resistance, and 112 accessions were susceptible (**Figure 1B**). Among the 91 resistant accessions, 63 were HR, including 39 hard and 24 soft winter wheat accessions (**Table 1**), and 28 were MR accessions including 19 hard and 9 soft winter wheat accessions (**Supplementary Table S2**). Among the 63 HR accessions, 35 accessions (18 hard and 17 soft winter wheat) showed complete resistance with a resistance score of 100% in at least two experiments (**Table 1**). The HR accessions distributed in 15 U.S. major winter wheat growing states, with most of them from these states where HF resistance is a major breeding objective including Kansas (10), Nebraska (10), Indiana (9), Oklahoma (8), and South Dakota (7) (**Table 1**). The results suggested a high frequency of HF resistant genotypes in the U.S. winter wheat. High and significant correlation coefficients of HF resistance scores for the accessions in the AM203 panel (0.83–0.93, *P* < 0.01) among the three experiments suggest high repeatability of the phenotypic data in this study.

### 3.3 HF resistance of wheat accessions from Iran

A set of 369 Iranian wheat accessions were screened for HF resistance in spring 2020. Among them, 107 accessions showed various levels of HF resistance including eight HR and 99 MR accessions, whereas the remaining 262 accessions were completely susceptible (**Figure 1C**). To purify the seeds from the 107 selected accessions, only 82 plants (accessions) successfully produced seeds. Further screening of the seedlings from the purified seeds confirmed only 29 (35.4%) accessions with various levels of HF resistance (**Figure 1C**), with four HR and 25 MR accessions (**Table 1**; **Supplementary Table S1**). Among the identified resistant accessions, PI 627466 showed consistently high resistance in both initial and confirmational HF screening experiments, and PI 627882, TAK-AB, and ZAGROS-R showed significant improvement in HF resistance after seed purification (**Figure 1D**, **Table 1**).

To demonstrate the possible heterogeneity in the original seed stock, susceptible plant was also recovered from ZAGROS, designated as ZAGROS-S. Its progeny remained completely susceptible in the HF test (**Figure 1D**), suggesting true heterogeneity of the original ZAGROS seed stock in terms of fly resistance. Seven lines (KARKHEH, PI 622247, DEHDASHT, PI 627987, PI 623908, PI 623905, and PI 627551) showed decreased HF resistance after seed purification, and two lines (PI 625250 and SABA) became completely susceptible (**Figure 1E**). In total, four HR and 25 MR accessions were confirmed with HF resistance using the purified seeds.

### 3.4 HF resistant accessions from other Asian countries

The Pakistani panel contains 176 wheat accessions. Only one accession (‘PARVAZ-94’) showed complete HF resistance (**Table 1**), the remaining accessions were completely susceptible. The resistance of PARVAZ-94 was confirmed by screening the purified seeds from a single resistant plant (**Table 1**).

A panel of 153 landraces and cultivars from East Asia including China, South Korea and Japan was also evaluated for HF resistance. Only one accession, ‘Chokwang’ from South Korea, showed complete HF resistance in both the initial screening and the second screening using the purified seeds from single resistant plants (**Table 1**). Two accessions, namely ‘Yangmai 4’ and ‘Suyang 7-2’ showed MR with HF resistance scores of 26.7% and 40.0%, respectively. All the other 150 accessions were completely susceptible. The results indicate a very low frequency of HF resistance sources in wheat from Pakistan and East Asia.

### 3.5 Geographic distribution of HF resistance sources

Among all evaluated wheat accessions, only 96 (3.8%) and 144 (5.8%) were HR and MR accessions, respectively (**Supplementary Table S3**). These resistant accessions are unevenly distributed in different wheat-growing countries. Among the 96 HR accessions, 78 (81.3%) were from the U.S., five from Iran, two from Russian and Azerbaijan, and one from Pakistan, Switzerland, Bulgaria, France, Croatia, South Korea, Former Soviet Union, Mexico, and New Zealand (**Figure 1F**; **Supplementary Table S3**). In addition, most MR accessions were also identified from the U.S. and Iran, with 44 (30.6%) and 36 (25.0%) MR accessions from U.S. and Iran, respectively (**Figure 1F**; **Supplementary Table S3**).

### 3.6 Reactions of the known HF resistance genes to HF biotype GP

For comparison, we also evaluated 39 wheat accessions that carry known HF-resistance genes. All these lines showed various levels of fly resistance except for ‘Dawson’, which carries *H1H2* (**Supplementary Table S4**). Among them, 14 accessions were completely resistant, 19 with HF resistance scores of 53–97%. Only ‘Java’ (*h4*), ‘Hamlet’ (*H21*), ‘KS89WGRC03’ (*H23*), ‘KS89WGRC06’ (*H24*), and ‘921696-H31’ (*H31*) showed MR reactions (30% to 44%) (**Supplementary Table S4**). These data suggest that most cultivars with known resistance genes are still highly effective against HF biotype GP.

## 4 Discussion

### 4.1 Extensive screening and novel HF resistant sources identification

In this study, we evaluated a comprehensive, representative collection of 2,496 wheat accessions for HF resistance, and identified 96 HR and 144 MR accessions from 104 countries. Therefore, this effort represents the most extensive search for novel HF resistant sources from bread wheat to date.

Previously, limited studies have been conducted to search for new sources of resistance genes to the predominant HF biotypes. El Bouhssini et al. (2008) screened 623 accessions of wheat and its wild relatives from 18 *Aegilops* species and 11 *Triticum* species for resistance to a highly virulent Syrian HF biotype and identified only 29 *Aegilops* accessions and four synthetic bread wheat accessions that were resistant to that biotype. Afterward, they screened additional 914 synthetic hexaploidy wheat accessions developed by the International Maize and Wheat Improvement Center (CIMMYT) for resistance to a HF population collected from northwest Syria and found 20 accessions with resistance scores of 40–100% (El Bouhssini et al., 2013). Bassi et al. (2019) screened 159 modern durum wheat cultivars and elite lines derived from eight Moroccan breeding programs for resistance to a Moroccan HF population and only identified six resistant accessions. Bouktila et al. (2005) found four resistant Tunisian durum and bread wheat varieties to a HF population from North Tunisia. In these studies, HF resistant sources were mainly identified from wheat relatives such as durum, *Aegilops* and other *Triticum* species.

Therefore, the current study extends these findings by conducting a comprehensive search for HF resistances specifically in a worldwide collection of bread wheat. To facilitate future usage of the identified resistant accessions in breeding programs, the identified resistant accessions were purified via single seed selection with progeny to be confirmed with resistance. The resulting resistant bread wheat lines, therefore, are poised to enhance breeding programs directly.

### 4.2 Geographic distribution and resistance correlation

The distribution of HR and MR wheat accessions revealed significant geographical variance. The identified HR wheat accessions were only from 13 countries with MR accessions from 46 countries (**Figure 1F**; **Supplementary Table S3**). Notably, our data do suggest that the frequencies of HF resistant accessions correlate with HF pressure in individual countries. The countries with most HR and MR accessions are those with HF as a major pest such as the U.S. (36.1%), Switzerland (10.3%), New Zealand (18.2%), Germany (11.1%), Portugal (18.2%), and Morocco (33.3%) (**Supplementary Table S3**) (CABI, 2021). This suggests that high HF selection pressure may exert natural selection from wheat with HF resistance.

Consistent with this hypothesis, high frequencies of wheat accessions were observed in Ancient Mediterranean countries, where wheat and HF might have been originated and coevolved for centuries (Stuart et al., 2012). Specifically, the countries with HF resistant wheat accessions include Azerbaijan (28.6%), Iraq (25%), and Syria (50%). Samples from those countries showed high frequencies of HF resistant accessions even with a small number of accessions screened from these countries (**Supplementary Table S3**). Our results also suggest that wheat HF resistance genes might occur before the formation or domestication of hexaploid wheat, which is supported by previous reports showing that many HF resistant accessions exist in durum, *Aegilops* and *Triticum* species (Bouktila et al., 2005; El Bouhssini et al., 2008, 2013; Bassi et al., 2019).

### 4.3 HF resistance within major U.S. wheat-producing areas

In the U.S., HF has been historically a major constraint for wheat production in the major U.S. wheat-production areas. Extensive research on HF resistance in wheat has been conducted for several decades with significant progress, including identification of 37 HF resistance genes and several resistance quantitative trait loci (QTL) (Xu et al., 2021), development of wheat near-isogenic lines (NILs) for various HF resistance genes (Dweikat et al., 1994, 1997; Kong et al., 2005), and establishment of relationship between wheat resistance genes and HF biotypes (Formusoh et al., 1996; Ratcliffe and Hatchett, 1997; Zantoko and Shukle, 1997). These achievements have significantly promoted the successful development of HF resistant cultivars in many U.S. wheat breeding programs.

In the Northwest U.S., Ando et al. (2018) identified 130 accessions resistant to a mixed population of HF biotypes from Washington and Idaho after screened a panel of 407 elite spring wheat cultivars and breeding lines, demonstrating a high frequency of HF resistant accessions in the U.S. spring wheat. The biotype GP is the most prevalent HF biotype in the U.S. Great Plains, the major hard winter wheat production area in the U.S (Sardesai et al., 2005; Chen et al., 2009; Garcés-Carrera et al., 2014; Tan et al., 2017). However, the status of HF resistance in winter wheat breeding programs remains unknown.

In this study, we screened a panel of 203 U.S. winter wheat accessions and identified 91 biotype GP resistant accessions (44.8%) with 63 HR accessions (**Table 1**), indicating HF resistance genes exist relatively widely in the U.S. hard and soft winter wheat cultivars. Many of these accessions identified in this study showed resistance not only to HF, but also to other wheat diseases such as Fusarium head blight (FHB) and powdery mildew (Jin et al., 2013; Liu et al., 2017). For example, Chisholm and SD05W148-1 exhibit high levels of resistance to powdery mildew (Liu et al., 2017); P04287A1-10, IL02-18228, M04-4715, IL00-8530, NI04420, SD05118, and MO040152 possess FHB resistance (Jin et al., 2013), and Roane, P03207A1-7, INW0411, M03-3616-C, and KY96C-0769-7-3 show high levels of resistance to both powdery mildew and FHB (Jin et al., 2013; Liu et al., 2017). The accessions with resistance to multiple diseases and insects can be directly used as the sources for developing cultivars resistant to multiple pests including HF.

### 4.4 Gene pyramiding and marker-assisted selection in HF resistance breeding

High level of resistance to HF in wheat might be conditioned by more than one gene. Gene pyramiding not only provides high levels of resistance to HF, but also improves the durability of wheat resistance. For most of the newly identified resistant accessions in the AM203 panel, only a few have been characterized by QTL mapping. ‘Clark’ is a soft winter wheat with complete HF resistance (resistance score of 100%) in this study (**Table 1**). Our previous study showed that resistance in Clark is conditioned by two new genes, *H34* with a major effect on chromosome 6B and a minor gene on chromosome 1A (Li et al., 2013). ‘SD06165’ is a hard winter wheat breeding line from South Dakota with two new HF resistance genes, a major effect gene *H35* on chromosome 3B and *H36* on 7A (Zhao et al., 2020). The combination of *H35* and *H36* provides consistently high levels of HF resistance (**Table 1**). In addition to SD06165, *H35* and *H36* also exist in the hard winter ‘Overland’ (**Table 1**) from Nebraska (Xu et al., 2021). Our results suggest that *H35* and *H36* have been deployed in some of the U.S. winter wheat cultivars. Breeder-friendly DNA markers have been developed for *H34, H35* and *H36* (Xu et al., 2021; Zhang et al., 2022).

The HR accessions identified in this study offer significant potential for enhancing HF resistance in wheat varieties both in the U.S. and globally. Advanced breeding techniques, such as marker-assisted gene pyramiding of resistance genes could significantly enhance the durability and level of HF resistance in new cultivars. The next steps will mainly focus on the following two aspects. Firstly, the development and utilization of molecular markers linked to the resistance genes identified in HF-resistant accessions is essential. While linkage mapping has been previously utilized, genome-wide association mapping (GWAS) presents a rapid and efficient alternative. GWAS can provide deeper insights into the genetic relationships among the HF-resistant sources discovered in this study. Additionally, it can help uncover new HF resistance genes and facilitate the creation of breeder-friendly markers associated with these genes. Secondly, pyramiding multiple HF resistance genes using marker-assisted backcrossing (MABC). This strategy systematically integrates various HF resistance genes into new cultivars to broaden and strengthen their resistance. MABC enhances breeding efficiency by enabling precise gene selection with molecular markers, minimizing linkage drag, and maximizing genetic gains. Additionally, using diverse sources for these genes reduces dependence on any single genetic line, thus reducing the risk of genetic bottlenecks.

### 4.5 Future directions and considerations for HF resistant source screening

Most accessions from the regions where HF is not a major pest also showed partial resistance. These accessions have not been selected for HF resistance during the breeding process; heterogeneity at resistance loci might be an important factor for the low resistance scores. Purification may improve their resistance levels. In this study, we have purified 415 accessions by recovering one resistant seedling for each resistant accession. Single seed progeny purification has resulted in significant improvement in HF resistance levels in nine wheat resistance accessions (**Figure 1D**), indicating that heterozygosity is likely responsible for lower levels of HF resistance in some of the wheat accessions. However, it is unexpected that 27 purified accessions decreased resistance scores or became completely susceptible (**Figure 3E**, **Supplementary Table S1**). Hessian fly resistance in wheat is also greatly impacted by environmental factors, especially temperatures (Liu et al., 2007; Brahmi et al., 2021). Escape of HF infestation in the initial screening and variation in screening environments might be partially responsible for the inversion from resistant to susceptible after seed purification.

## 5 Conclusion

In summary, this study identified a relatively large number of HF resistant accessions of common bread wheat. Potentially heterogeneous accessions were purified by single plant selection. A total of 96 HR and 144 MR accessions were confirmed using the purified seeds. Most of these HF resistant accessions have not been reported before, and some of them may carry new genes for HF resistance. The novel HF resistant sources identified here in common bread wheat should be useful for rapid development HF resistant cultivars and deployment of new HF resistance genes to minimize HF damage. We will increase the seeds of these accessions and deposit them to the USDA-ARS NSGC to make them publicly available.

## Data availability statement

The original contributions presented in the study are included in the article/**Supplementary Material**. Further inquiries can be directed to the corresponding authors.

## Ethics statement

The manuscript presents research on animals that do not require ethical approval for their study.

## Author contributions

YX: Writing – original draft, Conceptualization, Writing – review & editing, Formal analysis, Investigation. NG: Writing–original draft, Investigation. SH: Writing – original draft, Investigation. XX: Writing – original draft, Investigation. ZS: Writing – original draft, Investigation. DZ: Writing – original draft, Investigation. LZ: Writing – original draft, Investigation. XL: Writing – original draft, Investigation. M–SC: Writing – original draft, Investigation, Methodology. GB: Project administration, Supervision, Writing – original draft, Conceptualization, Writing – review & editing.

## References

[B1] Alipour H. Bihamta M. R. Mohammadi V. Peyghambari S. A. Bai G. Zhang G. (2017). Genotyping-by-sequencing (GBS) revealed molecular genetic diversity of Iranian wheat landraces and cultivars. Front. Plant Sci. 8. doi: 10.3389/fpls.2017.01293 PMC5583605 28912785

[B2] Ando K. Rynearson S. Muleta K. T. Gedamu J. Girma B. Bosque-Perez N. A.. (2018). Genome-wide associations for multiple pest resistances in a Northwestern United States elite spring wheat panel. PloS One 13, e0191305. doi: 10.1371/journal.pone.0191305 29415008 PMC5802848

[B3] Bassi F. Brahmi H. Sabraoui A. Amri A. Nsarellah N. Nachit M.. (2019). Genetic identification of loci for Hessian fly resistance in durum wheat. Mol. Breed. 39, 24. doi: 10.1007/s11032-019-0927-1

[B4] Berzonsky W. A. Ding H. Haley S. D. Harris M. O. Lamb R. J. McKenzie R.. (2003). Breeding wheat for resistance to insects. Plant Breed. Rev. 22, 221–296. doi: 10.1002/9780470650202.ch5

[B5] Bouktila D. Mezghani M. Marrakchi M. Makni H. (2005). Identification of wheat sources resistant to Hessian fly, *Mayetiola destructor* (*Diptera: Cecidomyiidae*) in Tunisia. Int. J. Agric. Biol. 7, 799–803.

[B6] Brahmi H. Lazraq A. Boulamtat R. El Fakhouri K. Bassi F. M. El Bouhssini M. (2021). Effect of temperature on the expression of resistance to Hessian fly (Diptera: Cecidomyiidae) in durum wheat cultivars. Phytoparasitica 49, 357–362. doi: 10.1007/s12600-020-00877-6

[B7] CABI (2021). “ *Mayetiola destructor* (Hessian fly),” in Invasive Species Compendium (CAB International, Wallingford, UK). Available at: www.cabi.org/isc.

[B8] Cambron S. E. Buntin G. D. Weisz R. Holland J. D. Flanders K. L. Schemerhorn B. J.. (2010). Virulence in Hessian fly (Diptera: Cecidomyiidae) field collections from the southeastern United States to 21 resistance genes in wheat. J. Economic Entomol. 103, 2229–2235. doi: 10.1603/EC10219 21309248

[B9] Chen M. S. Echegaray E. Whitworth R. J. Wang H. Y. Sloderbeck P. E. Knutson A.. (2009). Virulence analysis of Hessian fly populations from Texas, Oklahoma, and Kansas. J. Economic Entomol. 102, 774–780. doi: 10.1603/029.102.0239 19449660

[B10] Dweikat I. Ohm H. Mackenzie S. Patterson F. Cambron S. Ratcliffe R. (1994). Association of a DNA marker with Hessian fly resistance gene H9 in wheat. Theor. Appl. Genet. 89, 964–968. doi: 10.1007/BF00224525 24178111

[B11] Dweikat I. Ohm H. Patterson F. Cambron S. (1997). Identification of RAPD markers for 11 Hessian fly resistance genes in wheat. Theor. Appl. Genet. 94, 419–423. doi: 10.1007/s001220050431

[B12] El Bouhssini M. Nachit M. Valkoun J. Abdalla O. Rihawi F. (2008). Sources of resistance to Hessian fly (Diptera: Cecidomyiidae) in Syria identified among *Aegilops* species and synthetic derived bread wheat lines. Genet. Resour. Crop Evol. 55, 1215–1219. doi: 10.1007/s10722-008-9321-2

[B13] El Bouhssini M. Ogbonnaya F. Chen M. Lhaloui S. Rihawi F. Dabbous A. (2013). Sources of resistance in primary synthetic hexaploid wheat (*Triticum aestivum* L.) to insect pests: Hessian fly, Russian wheat aphid and Sunn pest in the fertile crescent. Genet. Resour. Crop Evol. 60, 621–627. doi: 10.1007/s10722-012-9861-3

[B14] Formusoh E. S. Hatchett J. H. Black W. C. Stuart J. J. (1996). Sex-linked inheritance of virulence against wheat resistance gene *H9* in the Hessian fly (Diptera: Cecidomyiidae). Ann. Entomological Soc. America 89, 428–434. doi: 10.1093/aesa/89.3.428

[B15] Garcés-Carrera S. Knutson A. Wang H. Giles K. L. Huang F. Whitworth R. J.. (2014). Virulence and biotype analyses of Hessian fly (Diptera: Cecidomyiidae) populations from Texas, Louisiana, and Oklahoma. J. Economic Entomol. 107, 417–423. doi: 10.1603/EC13372 24665728

[B16] Hatchett J. Gallun R. L. (1970). Genetics of the ability of the Hessian fly, *Mayetiola destructor*, to survive on wheats having different genes for resistance. Ann. Entomological Soc. America 63, 1400–1407. doi: 10.1093/aesa/63.5.1400

[B17] Jin F. Zhang D. Bockus W. W. Baenziger P. S. Carver B. Bai G. (2013). Fusarium head blight resistance in US winter wheat cultivars and elite breeding lines. Crop Sci. 53, 2006–2013. doi: 10.2135/cropsci2012.09.0531

[B18] Kong L. Cambron S. E. Ohm H. W. (2008). Hessian fly resistance genes *H16* and *H17* are mapped to a resistance gene cluster in the distal region of chromosome 1AS in wheat. Mol. Breed. 21, 183–194. doi: 10.1007/s11032-007-9119-5

[B19] Kong L. Ohm H. Cambron S. Williams C. (2005). Molecular mapping determines that Hessian fly resistance gene *H9* is located on chromosome 1A of wheat. Plant Breed. 124, 525–531. doi: 10.1111/j.1439-0523.2005.01139.x

[B20] Li C. L. Chen M. S. Chao S. M. Yu J. M. Bai G. H. (2013). Identification of a novel gene, *H34*, in wheat using recombinant inbred lines and single nucleotide polymorphism markers. Theor. Appl. Genet. 126, 2065–2071. doi: 10.1007/s00122-013-2118-5 23689741

[B21] Liu N. Bai G. Lin M. Xu X. Zheng W. (2017). Genome-wide association analysis of powdery mildew resistance in US winter wheat. Sci. Rep. 7, 1–11. doi: 10.1038/s41598-017-11230-z 28924158 PMC5603590

[B22] Liu X. Bai J. Huang L. Zhu L. Liu X. Weng N.. (2007). Gene expression of different wheat genotypes during attack by virulent and avirulent Hessian fly (*Mayetiola destructor*) larvae. J. Chem. Ecol. 33, 2171–2194. doi: 10.1007/s10886-007-9382-2 18058177

[B23] Liu X. M. Brown-Guedira G. L. Hatchett J. H. Owuoche J. O. Chen M. S. (2005). Genetic characterization and molecular mapping of a Hessian fly-resistance gene transferred from *T. turgidum* ssp *dicoccum* to common wheat. Theor. Appl. Genet. 111, 1308–1315. doi: 10.1007/s00122-005-0059-3 16136351

[B24] Martin-Sanchez J. Gomez-Colmenarejo M. Del Moral J. Sin E. Montes M. Gonzalez-Belinchon C.. (2003). A new Hessian fly resistance gene (*H30*) transferred from the wild grass *Aegilops triuncialis* to hexaploid wheat. Theor. Appl. Genet. 106, 1248–1255. doi: 10.1007/s00122-002-1182-z 12748776

[B25] Ratcliffe R. Hatchett J. (1997). “Biology and genetics of the Hessian fly and resistance in wheat,” in New developments in entomology. Ed. Bundari K. (India: Scientific Information Guild, Trivandrum), 47–56.

[B26] Sardesai N. Nemacheck J. A. Subramanyam S. Williams C. E. (2005). Identification and mapping of *H32*, a new wheat gene conferring resistance to Hessian fly. Theor. Appl. Genet. 111, 1167–1173. doi: 10.1007/s00122-005-0048-6 16160821

[B27] Shukle R. H. Cambron S. E. Moniem H. A. Schemerhorn B. J. Redding J. David Buntin G.. (2016). Effectiveness of genes for Hessian fly (Diptera: Cecidomyiidae) resistance in the southeastern United States. J. Economic Entomol. 109, 399–405. doi: 10.1093/jee/tov292 26468515

[B28] Shukle R. H. Subramanyam S. Saltzmann K. A. Williams C. E. (2010). Ultrastructural changes in the midguts of hessian fly larvae feeding on resistant wheat. J. Insect Physiol. 56, 754–760. doi: 10.1016/j.jinsphys.2010.01.005 20116382

[B29] Stuart J. J. Chen M.-S. Shukle R. Harris M. O. (2012). Gall midges (Hessian flies) as plant pathogens. Annu. Rev. Phytopathol. 50, 339–357. doi: 10.1146/annurev-phyto-072910-095255 22656645

[B30] Tan C. T. Yu H. J. Yang Y. Xu X. Y. Chen M. S. Rudd J. C.. (2017). Development and validation of KASP markers for the greenbug resistance gene *Gb7* and the Hessian fly resistance gene *H32* in wheat. Theor. Appl. Genet. 130, 1867–1884. doi: 10.1007/s00122-017-2930-4 28624908

[B31] Williams C. Collier C. Sardesai N. Ohm H. Cambron S. (2003). Phenotypic assessment and mapped markers for *H31*, a new wheat gene conferring resistance to Hessian fly (Diptera: Cecidomyiidae). Theor. Appl. Genet. 107, 1516–1523. doi: 10.1007/s00122-003-1393-y 12928782

[B32] Xu Y. La G. Fatima N. Liu Z. Zhang L. Zhao L.. (2021). Precise mapping of QTL for Hessian fly resistance in the hard winter wheat cultivar ‘Overland’. Theor. Appl. Genet. 134, 3951–3962. doi: 10.1007/s00122-021-03940-w 34471944

[B33] Zantoko L. Shukle R. H. (1997). Genetics of virulence in the hessian fly to resistance gene *H13* in wheat. J. Heredity 88, 120–123. doi: 10.1093/oxfordjournals.jhered.a023069

[B34] Zhang D. Bai G. Zhu C. Yu J. Carver B. F. (2010). Genetic diversity, population structure, and linkage disequilibrium in U.S. elite winter wheat. Plant Genome 3, 117–127. doi: 10.3835/plantgenome2010.03.0004

[B35] Zhang L. Xu Y. Chen M.-S. Su Z. Liu Y. Xu Y.. (2022). Identification of a major QTL for Hessian fly resistance in wheat cultivar ‘Chokwang’. Crop J. 10, 775–782. doi: 10.1016/j.cj.2021.08.004

[B36] Zhao L. Abdelsalam N. R. Xu Y. Chen M.-S. Feng Y. Kong L. (2020). Identification of two novel Hessian fly resistance genes *H35* and *H36* in a hard winter wheat line SD06165 Theor. Appl. Genet. 133, 2343–2353. doi: 10.1007/s00122-020-03602-3 32436021

