# Supplementary material for: Evaluating a worldwide wheat collection for resistance to Hessian fly biotype ‘Great Plains’
Source: Front Plant Sci. 2024 May 22;15:1402218. doi: 10.3389/fpls.2024.1402218 (PMC11155297; doi:10.3389/fpls.2024.1402218)
Supplement: Supplementary file 1 [file Table_1.docx]

**Evaluating a Worldwide Wheat Collection for Resistance to Hessian Fly Biotype ‘Great Plains’**

Yunfeng Xu^1,3*^, Nida Ghori^1^, Shabbir Hussain^1^, Xiaoting Xu^1^, Zhenqi Su^1^, Dadong Zhang^1^, Lanfei Zhao^1^, Xuming Liu^2^, Ming-Shun Chen^2^, Guihua Bai^2,1*^

^1^ Department of Agronomy, Kansas State University, Manhattan, KS 66506, U.S.A.

^2^ Hard Winter Wheat Genetics Research Unit, USDA-ARS, Manhattan, KS 66506, U.S.A.

^3^ Agricultural Genomics Institute at Shenzhen (AGIS), Chinese Academy of Agricultural Sciences (CAAS), Shenzhen 518000, China

^*^Corresponding authors: xuyunfeng@caas.cn; guihua.bai@usda.gov

**SUPPORTING INFORMATION**

Table S1. The newly identified moderately resistant accessions with Hessian fly resistance scores 1–50% from the wheat global collection.

| **No.** | **Panels** | **ID in panel** | **Accession ID** | **Accession Name** | **Country** | **Initial screen** | **Purified seeds** | **HF resistance** |
| --- | --- | --- | --- | --- | --- | --- | --- | --- |
| 1 | NSGC407 | NSGC293 | PI415182 | ZG 884/73 | Croatia | 16.3 | 40.0 | MR |
| 2 |  | NSGC333 | PI525282 | 1130 | Morocco | 25.8 | 38.5 | MR |
| 3 |  | NSGC368 | PI578040 | FL 8150-J9-K1 | United States | 70.0 | 37.5 | MR |
| 4 |  | NSGC377 | PI596273 | TX92D7374 | United States | 73.3 | 33.3 | MR |
| 5 |  | NSGC263 | PI361868 | 63/1-7-15-12 | Denmark | 15.4 | 23.1 | MR |
| 6 |  | NSGC119 | PI192808 | Tremes Arroxeado | Portugal | 63.9 | 22.2 | MR |
| 7 |  | NSGC387 | PI614012 | CIGM98.748-1 | Mexico | 37.1 | 16.7 | MR |
| 8 |  | NSGC302 | PI438962 | Grekum 105 | Kazakhstan | 33.3 | 16.7 | MR |
| 9 |  | NSGC216 | PI330558 | Coeruleum | United Kingdom | 20.0 | 15.8 | MR |
| 10 |  | NSGC247 | PI351650 | T-1716 | Switzerland | 35.7 | 15.4 | MR |
| 11 |  | NSGC092 | PI185358 | Bic 386 | Morocco | 13.0 | 15.0 | MR |
| 12 |  | NSGC401 | PI633876 | P921696 | United States | 56.7 | 12.5 | MR |
| 13 |  | NSGC141 | PI222677 | 1668 | Iran | 20.8 | 12.5 | MR |
| 14 |  | NSGC069 | PI117758 | 428 | Australia | 18.8 | 11.8 | MR |
| 15 |  | NSGC005 | CItr7635 | CI 7635 | Russian Federation | 39.2 | 11.1 | MR |
| 16 |  | NSGC063 | PI115838 | 4453/31 | Germany | 25.0 | 11.1 | MR |
| 17 |  | NSGC206 | PI286544 | Colorado | Ecuador | 11.7 | 10.5 | MR |
| 18 |  | NSGC017 | CItr14204 | 2821-3B-1B-2B-5T | Colombia | 10.0 | 10.0 | MR |
| 19 |  | NSGC228 | PI345408 | 407-IV/60 | Bosnia and Herzegovina | 10.0 | 10.0 | MR |
| 20 |  | NSGC347 | PI559544 | KM 964-90 | Czech Republic | 8.3 | 9.1 | MR |
| 21 |  | NSGC400 | PI632399 | Richland | United States | 6.3 | 7.1 | MR |
| 22 |  | NSGC394 | PI619381 | NWX008094 | United States | 41.8 | 5.9 | MR |
| 23 |  | NSGC171 | PI254043 | No name 4 - Europe | Europe | 35.4 | 5.9 | MR |
| 24 |  | NSGC204 | PI283872 | Arawa | New Zealand | 14.2 | 5.9 | MR |
| 25 |  | NSGC026 | CItr15090 | Hard Red Calcutta | Canada | 8.3 | 5.9 | MR |
| 26 |  | NSGC023 | CItr14400 | 1957/3/18 | Peru | 23.2 | 5.6 | MR |
| 27 |  | NSGC197 | PI278545 | Aleppo 28 | Syria | 10.8 | 5.6 | MR |
| 28 |  | NSGC071 | PI124739 | C 10854 | Kenya | 10.0 | 5.6 | MR |
| 29 |  | NSGC341 | PI532255 | Mufsegha | Oman | 9.1 | 5.6 | MR |
| 30 |  | NSGC038 | PI8813 | Kurd | Iraq | 47.5 | 5.3 | MR |
| 31 |  | NSGC091 | PI184602 | 3CX3C | Spain | 11.1 | 5.3 | MR |
| 32 |  | NSGC225 | PI344017 | Maia do Vale | Angola | 10.0 | 5.3 | MR |
| 33 |  | NSGC124 | PI197250 | 9934 | Ethiopia | 8.3 | 5.3 | MR |
| 34 |  | NSGC193 | PI278405 | Barbela Grosso | Portugal | 7.9 | 5.3 | MR |
| 35 |  | NSGC149 | PI225424 | 63-569 | Uruguay | 5.6 | 5.3 | MR |
| 36 |  | NSGC146 | PI225271 | 85 | Iran | 22.1 | 5.0 | MR |
| 37 |  | NSGC142 | PI223151 | C.C.C. | Jordan | 8.3 | 5.0 | MR |
| 38 | NSGC1188 | NSGC0099 | PI65564 | Cologna Veneto | Italy | 33.3 | 50.0 | MR |
| 39 |  | NSGC0845 | PI596273 | TX92D7374 | United States | 62.5 | 41.2 | MR |
| 40 |  | NSGC0842 | PI593646 | NE91651 | United States | 62.5 | 40.0 | MR |
| 41 |  | NSGC0707 | PI476765 | 536 IIc-72 | Former Soviet Union | 16.7 | 37.5 | MR |
| 42 |  | NSGC0091 | PI57162 | CI 7109 | Georgia | 30.0 | 35.3 | MR |
| 43 |  | NSGC0838 | PI592481 | HBF0214-126 | United States | 90.0 | 33.3 | MR |
| 44 |  | NSGC0364 | PI285896 | Akakkowa Aka | Japan | 22.2 | 33.3 | MR |
| 45 |  | NSGC0834 | PI592439 | HBE0363-134 | United States | 86.7 | 31.6 | MR |
| 46 |  | NSGC0031 | CItr13225 | III-54-62 | United States | 57.1 | 30.0 | MR |
| 47 |  | NSGC0841 | PI592494 | HBF0303-152 | United States | 100.0 | 29.4 | MR |
| 48 |  | NSGC0331 | PI278539 | Aleppo 10 | Syria | 16.7 | 25.0 | MR |
| 49 |  | NSGC1148 | PI627905 | IWA8604142 | Iran | 5.6 | 25.0 | MR |
| 50 |  | NSGC0095 | PI57988 | 175 | India | 6.7 | 22.2 | MR |
| 51 |  | NSGC0108 | PI84527 | 176 | Uzbekistan | 33.3 | 20.0 | MR |
| 52 |  | NSGC0647 | PI415192 | ZG 4240/73 | Croatia | 14.3 | 20.0 | MR |
| 53 |  | NSGC0464 | PI350046 | 650-IV/6 | Serbia | 33.3 | 18.8 | MR |
| 54 |  | NSGC0145 | PI157949 | Romano | Italy | 25.0 | 18.8 | MR |
| 55 |  | NSGC0204 | PI191506 | Rosafe Argentino | United Kingdom | 11.8 | 18.8 | MR |
| 56 |  | NSGC0512 | PI351461 | 62/9 (ID) | Italy | 40.0 | 18.2 | MR |
| 57 |  | NSGC0898 | PI621251 | IWA8607162 | Iran | 100.0 | 16.7 | MR |
| 58 |  | NSGC0712 | PI478009 | HR 53 | United States | 25.0 | 16.7 | MR |
| 59 |  | NSGC1116 | PI627424 | IWA8602355 | Iran | 16.7 | 16.7 | MR |
| 60 |  | NSGC0218 | PI192582 | Plantahof 3 | Switzerland | 33.3 | 14.3 | MR |
| 61 |  | NSGC0207 | PI192030 | Roemer | Germany | 13.3 | 13.3 | MR |
| 62 |  | NSGC0229 | PI195545 | Hohenauer | Austria | 5.6 | 12.5 | MR |
| 63 |  | NSGC0570 | PI362189 | Yung Kwang | South Korea | 100.0 | 11.8 | MR |
| 64 |  | NSGC1179 | PI636141 | PI560596-sel-wcors | United States | 22.2 | 11.8 | MR |
| 65 |  | NSGC0181 | PI184168 | Crvenica | Serbia | 14.3 | 11.8 | MR |
| 66 |  | NSGC0102 | PI68273 | 100 | Azerbaijan | 10.0 | 11.8 | MR |
| 67 |  | NSGC0349 | PI284531 | Acciaio | Italy | 25.0 | 9.1 | MR |
| 68 |  | NSGC1161 | PI628118 | IWA8604712 | Iran | 50.0 | 7.7 | MR |
| 69 |  | NSGC0749 | PI519267 | Transelvania 1 | Romania | 37.5 | 7.7 | MR |
| 70 |  | NSGC0068 | CItr16762 | CAR 835 | Chile | 14.3 | 7.7 | MR |
| 71 |  | NSGC0046 | CItr15267 | Marzotto | Italy | 50.0 | 7.1 | MR |
| 72 |  | NSGC0561 | PI361980 | T 2339/65 | Romania | 20.0 | 7.1 | MR |
| 73 |  | NSGC0757 | PI559524 | KM 600-3-90 | Czech Republic | 16.7 | 7.1 | MR |
| 74 |  | NSGC1190 | PI639107 | KW981718h0024 | United States | 14.3 | 7.1 | MR |
| 75 |  | NSGC1157 | PI628047 | IWA8604571 | Iran | 76.9 | 6.7 | MR |
| 76 |  | NSGC1044 | PI625450 | IWA8611239 | Iran | 33.3 | 6.7 | MR |
| 77 |  | NSGC0100 | PI68236 | 413 | Azerbaijan | 20.0 | 6.7 | MR |
| 78 |  | NSGC0183 | PI184185 | Ranka | Bosnia and Herzegovina | 55.6 | 6.3 | MR |
| 79 |  | NSGC0035 | CItr14011 | SD 6718 | United States | 28.6 | 6.3 | MR |
| 80 |  | NSGC0144 | PI157906 | GR 2 | Italy | 25.0 | 6.3 | MR |
| 81 |  | NSGC0070 | CItr16860 | CAR 933 | Chile | 18.2 | 6.3 | MR |
| 82 |  | NSGC0509 | PI351426 | Cloches 32 | France | 16.7 | 6.3 | MR |
| 83 |  | NSGC0019 | CItr11366 | Varna 37 | Bulgaria | 11.1 | 6.3 | MR |
| 84 |  | NSGC0274 | PI262605 | Karagach | Turkmenistan | 100.0 | 5.9 | MR |
| 85 |  | NSGC0836 | PI592445 | HBF0302-148 | United States | 100.0 | 5.9 | MR |
| 86 |  | NSGC0895 | PI621237 | IWA8607130 | Iran | 50.0 | 5.9 | MR |
| 87 |  | NSGC1124 | PI627593 | IWA8602986 | Iran | 37.5 | 5.6 | MR |
| 88 |  | NSGC0093 | PI57171 | CI 7118 | Georgia | 25.0 | 5.6 | MR |
| 89 |  | NSGC0246 | PI220843 | Gandum | Afghanistan | 14.3 | 5.6 | MR |
| 90 |  | NSGC0307 | PI278249 | Ostmarker | Germany | 10.0 | 5.6 | MR |
| 91 |  | NSGC1020 | PI623468 | IWA8606075 | Iran | 10.0 | 5.6 | MR |
| 92 | IRAN369 | IRAN366 | NA | KARKHEH | Iran | 89.3 | 43.8 | MR |
| 93 |  | IRAN162 | NA | PI622247 | Iran | 54.5 | 35.3 | MR |
| 94 |  | IRAN295 | NA | DEHDASHT | Iran | 78.3 | 29.4 | MR |
| 95 |  | IRAN081 | PI627356 | NA | Iran | 4.2 | 29.4 | MR |
| 96 |  | IRAN101 | PI627856 | NA | Iran | 2.6 | 29.4 | MR |
| 97 |  | IRAN125 | PI627987 | NA | Iran | 58.8 | 22.2 | MR |
| 98 |  | IRAN219 | PI623908 | NA | Iran | 78.3 | 21.4 | MR |
| 99 |  | IRAN218 | PI623905 | NA | Iran | 48.3 | 20.0 | MR |
| 100 |  | IRAN221 | PI623953 | NA | Iran | 5.9 | 20.0 | MR |
| 101 |  | IRAN055 | PI627072 | NA | Iran | 8.7 | 18.8 | MR |
| 102 |  | IRAN197 | PI623266 | NA | Iran | 5.3 | 18.8 | MR |
| 103 |  | IRAN024 | PI625263 | NA | Iran | 11.1 | 16.7 | MR |
| 104 |  | IRAN059 | PI627189 | NA | Iran | 13.0 | 15.4 | MR |
| 105 |  | IRAN263 | PI624983 | NA | Iran | 8.3 | 15.4 | MR |
| 106 |  | IRAN049 | PI626932 | NA | Iran | 2.7 | 14.3 | MR |
| 107 |  | IRAN226 | PI624251 | NA | Iran | 8.1 | 13.3 | MR |
| 108 |  | IRAN176 | PI622063 | NA | Iran | 33.3 | 12.5 | MR |
| 109 |  | IRAN109 | PI627760 | NA | Iran | 13.3 | 12.5 | MR |
| 110 |  | IRAN196 | PI622930 | NA | Iran | 20.0 | 11.1 | MR |
| 111 |  | IRAN111 | PI627551 | NA | Iran | 43.2 | 10.5 | MR |
| 112 |  | IRAN047 | PI626923 | NA | Iran | 28.1 | 9.1 | MR |
| 113 |  | IRAN070 | PI627036 | NA | Iran | 8.1 | 6.7 | MR |
| 114 |  | IRAN189 | PI622319 | NA | Iran | 2.6 | 6.7 | MR |
| 115 |  | IRAN019 | PI625081 | NA | Iran | 9.1 | 6.3 | MR |
| 116 |  | IRAN201 | PI623338 | NA | Iran | 2.7 | 5.9 | MR |

Table S2 The accession names, classes and states the accessions from, and Hessian fly resistance scores for the 203 accessions from the U.S. AM203 panel screened in fall 2013, and spring and fall of 2019 greenhouse experiments.

| **ID in panel** | **Accession ID** | **Accession Name** | **Class** | **State** | **Nursery/ID** | **Fall 2013** | **Spring 2019** | **Fall 2019** | **Mean** | **HF resistance** |
| --- | --- | --- | --- | --- | --- | --- | --- | --- | --- | --- |
| AM010 | AM010 | Duster | HRW | OK | PI644016 | 100.0 | 100.0 | 100.0 | 100.0 | HR |
| AM029 | AM029 | SD06173 | HRW | SD | NRPN | 100.0 | 100.0 | 100.0 | 100.0 | HR |
| AM034 | AM034 | HV9W03-696R-1 | HRW | KS | SRPN | 100.0 | 100.0 | 100.0 | 100.0 | HR |
| AM090 | AM090 | NE06619 | HRW | NE | RGON | 100.0 | 100.0 | - | 100.0 | HR |
| AM095 | AM095 | OH02-7217 | SRW | OH | UESRWWN | 100.0 | 100.0 | 100.0 | 100.0 | HR |
| AM108 | AM108 | TX06A001431 | HRW | TX | RGON | 100.0 | 100.0 | 100.0 | 100.0 | HR |
| AM115 | AM115 | SD07204 | HRW | SD | RGON | 100.0 | 100.0 | 100.0 | 100.0 | HR |
| AM119 | AM119 | KY96C-0769-7-3 | SRW | KY | UESRWWN | 100.0 | 100.0 | 100.0 | 100.0 | HR |
| AM120 | AM120 | P03207A1-7 | SRW | IN | UESRWWN | 100.0 | 100.0 | 100.0 | 100.0 | HR |
| AM126 | AM126 | Roane | SRW | VA | PI612958 | 100.0 | 100.0 | 100.0 | 100.0 | HR |
| AM130 | AM130 | KS010514-9TM-10 | HRW | KS | RGON | 100.0 | 100.0 | - | 100.0 | HR |
| AM132 | AM132 | INW0411 | SRW | IN | UESRWWN | 100.0 | 100.0 | 100.0 | 100.0 | HR |
| AM138 | AM138 | Branson | SRW | IN | UESRWWN | 100.0 | 100.0 | 100.0 | 100.0 | HR |
| AM140 | AM140 | IL02-18228 | SRW | IL | UESRWWN | 100.0 | 100.0 | 100.0 | 100.0 | HR |
| AM141 | AM141 | KS07HW117 | HWW | KS | RGON | 100.0 | 100.0 | 100.0 | 100.0 | HR |
| AM145 | AM145 | IL02-19463 | SRW | IL | UESRWWN | 100.0 | 100.0 | 100.0 | 100.0 | HR |
| AM149 | AM149 | M03-3616-C | SRW | IN | USSRWWN | 100.0 | 100.0 | 100.0 | 100.0 | HR |
| AM150 | AM150 | W98007V1 | SRW | SC | USSRWWN | 100.0 | 100.0 | 100.0 | 100.0 | HR |
| AM170 | AM170 | D04-5012 | SRW | AR | USSRWWN | 100.0 | 100.0 | 100.0 | 100.0 | HR |
| AM188 | AM188 | OK06848W | HRW | OK | OSU | 100.0 | 100.0 | 100.0 | 100.0 | HR |
| AM200 | AM200 | P04287A1-10 | SRW | IN | USSRWWN | 100.0 | 100.0 | 100.0 | 100.0 | HR |
| AM201 | AM201 | M04_4715 | SRW | IN | USSRWWN | - | - | 100.0 | 100.0 | HR |
| AM139 | AM139 | IL00-8530 | SRW | IL | UESRWWN | 100.0 | 100.0 | 95.0 | 98.3 | HR |
| AM155 | AM155 | W98008J1 | SRW | SC | USSRWWN | 100.0 | 100.0 | 95.0 | 98.3 | HR |
| AM040 | AM040 | NW04Y2188 | HWW | NE | NRPN | 100.0 | 100.0 | 90.0 | 96.7 | HR |
| AM067 | AM067 | KS05HW121-2 | HWW | KS | SRPN | 100.0 | 100.0 | 89.5 | 96.5 | HR |
| AM157 | AM157 | OK06210 | HRW | OK | OSU | 100.0 | 100.0 | 84.4 | 94.8 | HR |
| AM146 | AM146 | Mocha exp. | SRW | OH | UESRWWN | 100.0 | 100.0 | 80.0 | 93.3 | HR |
| AM103 | AM103 | OH03-41-45 | SRW | OH | UESRWWN | 100.0 | 85.0 | 94.7 | 93.2 | HR |
| AM082 | AM082 | TX04M410164 | HRW | TX | SRPN | 100.0 | 100.0 | 76.5 | 92.2 | HR |
| AM015 | AM015 | NE04424 | HRW | NE | SRPN | 100.0 | 100.0 | 75.0 | 91.7 | HR |
| AM031 | AM031 | NE05548 | HRW | NE | NRPN | 100.0 | 100.0 | 75.0 | 91.7 | HR |
| AM019 | AM019 | SD06165 | HRW | SD | NRPN | 100.0 | 78.6 | 94.4 | 91.0 | HR |
| AM063 | AM063 | NE02558 | HRW | NE | NRPN | 100.0 | 85.0 | 85.0 | 90.0 | HR |
| AM151 | AM151 | Arena exp. | SRW | OH | UESRWWN | 100.0 | 100.0 | 70.0 | 90.0 | HR |
| AM009 | AM009 | NI04420 | HRW | NE | NRPN | 96.4 | 85.0 | 85.0 | 88.8 | HR |
| AM062 | AM062 | SD05118 | HRW | SD | NRPN | 100.0 | 89.5 | 75.0 | 88.2 | HR |
| AM098 | AM098 | KS020304K~3 | HRW | KS | RGON | 100.0 | 100.0 | 60.0 | 86.7 | HR |
| AM106 | AM106 | NE06436 | HRW | NE | RGON | 100.0 | 100.0 | 60.0 | 86.7 | HR |
| AM197 | AM197 | MO040152 | SRW | MO | UESRWWN | 85.0 | 85.0 | 88.9 | 86.3 | HR |
| AM069 | AM069 | KS970187-1-10 | HRW | KS | SRPN | 100.0 | 61.1 | 88.9 | 83.3 | HR |
| AM085 | AM085 | Chisholm | HRW | OK | PI486219 | 100.0 | 100.0 | 40.0 | 80.0 | HR |
| AM142 | AM142 | NE06549 | HRW | NE | RGON | 75.0 | 90.0 | 75.0 | 80.0 | HR |
| AM041 | AM041 | NE05549 | HRW | NE | NRPN | 100.0 | 50.0 | 80.0 | 76.7 | HR |
| AM164 | AM164 | OK06345 | HRW | OK | OSU | - | 70.0 | 80.0 | 75.0 | HR |
| AM003 | AM003 | KS05HW136-3 | HWW | KS | SRPN | 100.0 | 70.6 | 52.9 | 74.5 | HR |
| AM101 | AM101 | TX06A001376 | HRW | TX | RGON | 95.0 | 89.5 | 37.9 | 74.1 | HR |
| AM039 | AM039 | SD03164-1 | HRW | SD | NRPN | 100.0 | 35.0 | 85.0 | 73.3 | HR |
| AM129 | AM129 | SD05W148-1 | HWW | SD | RGON | 75.0 | 90.0 | 50.0 | 71.7 | HR |
| AM025 | AM025 | HV9W03-539R | HRW | KS | SRPN | 100.0 | 66.7 | 45.0 | 70.6 | HR |
| AM159 | AM159 | G69202 | SRW | IN | UESRWWN | 0.0 | 100.0 | 100.0 | 66.7 | HR |
| AM194 | AM194 | GA991371-6E13 | SRW | GA | USSRWWN | 65.0 | 65.0 | 65.0 | 65.0 | HR |
| AM180 | AM180 | OK05134 | HRW | OK | OSU | 100.0 | 20.0 | 66.7 | 62.2 | HR |
| AM131 | AM131 | N02Y5117 | HRW | NE | RGON | 100.0 | 45.0 | 30.0 | 58.3 | HR |
| AM178 | AM178 | GA991336-6E9 | SRW | GA | USSRWWN | 15.0 | 70.0 | 89.5 | 58.2 | HR |
| AM058 | AM058 | KS970093-8-9-#1 | HRW | KS | SRPN | 90.0 | 42.1 | 40.0 | 57.4 | HR |
| AM171 | AM171 | G59160 | SRW | IN | USSRWWN | 100.0 | 45.0 | 25.0 | 56.7 | HR |
| AM147 | AM147 | Pioneer Brand 26R61 | SRW | IN | USSRWWN | 100.0 | 15.0 | 52.6 | 55.9 | HR |
| AM073 | AM073 | SD05210 | HRW | SD | NRPN | 18.2 | 100.0 | 45.0 | 54.4 | HR |
| AM033 | AM033 | Trego | HWW | KS | PI612576 | 5.0 | 75.0 | 80.0 | 53.3 | HR |
| AM059 | AM059 | CO03W239 | HWW | CO | SRPN | 60.0 | 75.0 | 25.0 | 53.3 | HR |
| AM113 | AM113 | OK05511 | HRW | OK | RGON | 100.0 | 30.0 | 30.0 | 53.3 | HR |
| AM054 | AM054 | OK05903C | HRW | OK | OSU | 100.0 | 10.0 | 45.0 | 51.7 | HR |
| AM114 | AM114 | SD07W041 | HWW | SD | RGON | - | 50.0 | 50.0 | 50.0 | MR |
| AM105 | AM105 | HV9W05-881R | HRW | KS | RGON | 5.0 | 80.0 | 55.0 | 46.7 | MR |
| AM192 | AM192 | P03112A1-7-14 | SRW | IN | USSRWWN | 100.0 | 30.0 | 0.0 | 43.3 | MR |
| AM153 | AM153 | VA05W-258 | SRW | VA | USSRWWN | 25.0 | 50.0 | 50.0 | 41.7 | MR |
| AM181 | AM181 | OK06313 | HRW | OK | OSU | 100.0 | 20.0 | 0.0 | 40.0 | MR |
| AM165 | AM165 | OK06319 | HRW | OK | OSU | 50.0 | 25.0 | 35.0 | 36.7 | MR |
| AM096 | AM096 | MD99W483-06-9 | SRW | MD | UESRWWN | 72.2 | 5.0 | 27.8 | 35.0 | MR |
| AM021 | AM021 | NI04427 | HRW | NE | NRPN | 16.0 | 40.0 | 40.0 | 32.0 | MR |
| AM026 | AM026 | CO03064 | HRW | CO | SRPN | 0.0 | 77.8 | 15.0 | 30.9 | MR |
| AM134 | AM134 | NYCalR-L | SRW |  | UESRWWN | 75.0 | 15.0 | 0.0 | 30.0 | MR |
| AM013 | AM013 | AP04T8211 | HRW | KS | SRPN | 76.9 | 10.0 | 0.0 | 29.0 | MR |
| AM128 | AM128 | LA02-923 | SRW | IN | UESRWWN | 0.0 | 20.0 | 45.0 | 21.7 | MR |
| AM017 | AM017 | OK03825-5403-6 | HRW | OK | SRPN | 52.4 | 5.0 | 5.0 | 20.8 | MR |
| AM089 | AM089 | KS010957K~4 | HRW | KS | RGON | 0.0 | 20.0 | 25.0 | 15.0 | MR |
| AM202 | AM202 | GA991227-6A33 | SRW | GA | USSRWWN | 11.8 | 11.8 | 11.8 | 11.8 | MR |
| AM005 | AM005 | KS980554-12-~9 | HRW | KS | SRPN | 7.4 | 20.0 | 0.0 | 9.1 | MR |
| AM094 | AM094 | MO011126 | SRW | MO | UESRWWN | 25.0 | 0.0 | 0.0 | 8.3 | MR |
| AM161 | AM161 | LA01138D-52 | SRW | LA | USSRWWN | 0.0 | 10.0 | 15.0 | 8.3 | MR |
| AM020 | AM020 | NX03Y2489 | HWW | NE | NRPN | 14.3 | 0.0 | 6.3 | 6.8 | MR |
| AM046 | AM046 | HV9W02-942R | HRW | KS | SRPN | 10.0 | 10.0 | 0.0 | 6.7 | MR |
| AM068 | AM068 | T153 | HRW | KS | SRPN | 0.0 | 10.0 | 10.0 | 6.7 | MR |
| AM117 | AM117 | TXHT001F8-CS06/325-PRE07/75 | HRW | TX | RGON | 0.0 | 10.0 | 9.5 | 6.5 | MR |
| AM160 | AM160 | USG 3555 | SRW | VA | USSRWWN | 0.0 | 5.0 | 11.1 | 5.4 | MR |
| AM012 | AM012 | Scout 66 | HRW | NE | Citr13996 | 0.0 | 5.0 | 10.5 | 5.2 | MR |
| AM056 | AM056 | KS05HW15-2 | HWW | KS | SRPN | 0.0 | 10.0 | 5.0 | 5.0 | MR |
| AM203 | AM203 | OK05128 | HRW | OK | OSU | 5.0 | 5.0 | 5.0 | 5.0 | MR |
| AM055 | AM055 | Century | HRW | OK | PI502912 | 0.0 | 5.0 | 5.0 | 3.3 | MR |
| AM065 | AM065 | Fuller | HRW | KS | certified | 5.0 | 5.0 | 0.0 | 3.3 | MR |
| AM093 | AM093 | TXHT006F8-CS06/472-STA34 | HRW | TX | RGON | 65.0 | 0.0 | 0.0 | 21.7 | S |
| AM043 | AM043 | OK03716W | HRW | OK | OSU | 0.0 | 0.0 | 55.0 | 18.3 | S |
| AM118 | AM118 | CO04W210 | HWW | CO | RGON | 50.0 | 0.0 | 0.0 | 16.7 | S |
| AM047 | AM047 | NE05430 | HRW | NE | SRPN | 0.0 | 26.3 | 0.0 | 8.8 | S |
| AM152 | AM152 | Coker 9553 | SRW | IN | USSRWWN | 20.0 | 0.0 | 0.0 | 6.7 | S |
| AM173 | AM173 | OK06528 | HRW | OK | OSU | 20.0 | 0.0 | 0.0 | 6.7 | S |
| AM184 | AM184 | VA04W-259 | SRW | VA | USSRWWN | 0.0 | 20.0 | 0.0 | 6.7 | S |
| AM186 | AM186 | GA991209-6E33 | SRW | GA | USSRWWN | 0.0 | 20.0 | 0.0 | 6.7 | S |
| AM061 | AM061 | Jerry | HRW | ND | PI632433 | 0.0 | 15.0 | 0.0 | 5.0 | S |
| AM091 | AM091 | MTS04120 | HRW | MT | RGON | 0.0 | 15.0 | 0.0 | 5.0 | S |
| AM125 | AM125 | NE06472 | HRW | NE | RGON | 15.0 | 0.0 | 0.0 | 5.0 | S |
| AM183 | AM183 | M04*5109 | SRW | IN | UESRWWN | 15.0 | 0.0 | 0.0 | 5.0 | S |
| AM035 | AM035 | NE05426 | HRW | NE | SRPN | 0.0 | 10.5 | 0.0 | 3.5 | S |
| AM045 | AM045 | AP06T3832 | HRW | KS | SRPN | 0.0 | 10.5 | 0.0 | 3.5 | S |
| AM081 | AM081 | NE05496 | HRW | NE | SRPN | 0.0 | 10.0 | 0.0 | 3.3 | S |
| AM102 | AM102 | VA03W-412 | SRW | VA | UESRWWN | 0.0 | 10.0 | 0.0 | 3.3 | S |
| AM024 | AM024 | AP05T2413 | HRW | KS | SRPN | 6.3 | 0.0 | 0.0 | 2.1 | S |
| AM006 | AM006 | KS980512-2-2 | HRW | KS | SRPN | 0.0 | 5.6 | 0.0 | 1.9 | S |
| AM016 | AM016 | CO02W237 | HWW | CO | SRPN | 5.3 | 0.0 | 0.0 | 1.8 | S |
| AM030 | AM030 | NX04Y2107 | HWW | NE | NRPN | 0.0 | 5.3 | 0.0 | 1.8 | S |
| AM064 | AM064 | MT0495 | HRW | MT | NRPN | 0.0 | 5.0 | 0.0 | 1.7 | S |
| AM072 | AM072 | SD06W117 | HRW | SD | NRPN | 0.0 | 0.0 | 5.0 | 1.7 | S |
| AM086 | AM086 | Guymon | HRW | OK | PI643133 | 0.0 | 0.0 | 5.0 | 1.7 | S |
| AM123 | AM123 | SD07220 | HRW | SD | RGON | 0.0 | 5.0 | 0.0 | 1.7 | S |
| AM156 | AM156 | OK05122 | HRW | OK | OSU | 0.0 | 0.0 | 5.0 | 1.7 | S |
| AM187 | AM187 | G41732 | SRW | IN | USSRWWN | 0.0 | 5.0 | 0.0 | 1.7 | S |
| AM004 | AM004 | T158 | HRW | KS | SRPN | 3.8 | 0.0 | 0.0 | 1.3 | S |
| AM001 | AM001 | Atlas66 | SRW | NC | Citr12561 | 0.0 | 0.0 | 0.0 | 0.0 | S |
| AM002 | AM002 | OK04505 | HRW | OK | SRPN | 0.0 | 0.0 | 0.0 | 0.0 | S |
| AM007 | AM007 | TX04M410211 | HRW | TX | SRPN | 0.0 | 0.0 | 0.0 | 0.0 | S |
| AM008 | AM008 | N98L20040-44 | HRW | NE | NRPN | 0.0 | 0.0 | 0.0 | 0.0 | S |
| AM011 | AM011 | OK02522W | HRW | OK | SRPN | 0.0 | 0.0 | 0.0 | 0.0 | S |
| AM014 | AM014 | HV9W96-1271R-1 | HRW | KS | SRPN | 0.0 | 0.0 | 0.0 | 0.0 | S |
| AM018 | AM018 | TX04V075080 | HRW | TX | SRPN | 0.0 | 0.0 | 0.0 | 0.0 | S |
| AM022 | AM022 | Endurance | HRW | OK | PI639233 | 0.0 | 0.0 | 0.0 | 0.0 | S |
| AM023 | AM023 | TAM-107 | HRW | TX | PI495594 | 0.0 | 0.0 | 0.0 | 0.0 | S |
| AM027 | AM027 | TX02A0252 | HRW | TX | SRPN | 0.0 | 0.0 | 0.0 | 0.0 | S |
| AM028 | AM028 | Kharkof | HRW | Russia | NRPN | 0.0 | 0.0 | 0.0 | 0.0 | S |
| AM032 | AM032 | Deliver | HRW | OK | PI639232 | 0.0 | 0.0 | 0.0 | 0.0 | S |
| AM036 | AM036 | CO03W054 | HWW | CO | SRPN | 0.0 | 0.0 | 0.0 | 0.0 | S |
| AM037 | AM037 | TX03A0148 | HRW | TX | SRPN | 0.0 | 0.0 | 0.0 | 0.0 | S |
| AM038 | AM038 | Antelope | HWW | NE | PI633910 | 0.0 | 0.0 | 0.0 | 0.0 | S |
| AM042 | AM042 | OK Bullet | HRW | OK | PI642415 | 0.0 | 0.0 | 0.0 | 0.0 | S |
| AM044 | AM044 | OK00514-05806 | HRW | OK | SRPN | 0.0 | 0.0 | 0.0 | 0.0 | S |
| AM048 | AM048 | CO03W139 | HWW | CO | SRPN | 0.0 | 0.0 | 0.0 | 0.0 | S |
| AM049 | AM049 | TX03A0563 | HRW | TX | SRPN | 0.0 | 0.0 | 0.0 | 0.0 | S |
| AM050 | AM050 | Wesley | HRW | NE | PI605742 | 0.0 | 0.0 | 0.0 | 0.0 | S |
| AM051 | AM051 | NE02533 | HRW | NE | NRPN | 0.0 | 0.0 | 0.0 | 0.0 | S |
| AM052 | AM052 | NE05569 | HRW | NE | NRPN | 0.0 | 0.0 | 0.0 | 0.0 | S |
| AM053 | AM053 | Overley | HRW | KS | certified | 0.0 | 0.0 | 0.0 | 0.0 | S |
| AM057 | AM057 | T151 | HRW | KS | SRPN | 0.0 | 0.0 | 0.0 | 0.0 | S |
| AM060 | AM060 | TX04A001246 | HRW | TX | SRPN | 0.0 | 0.0 | 0.0 | 0.0 | S |
| AM066 | AM066 | OK03522 | HRW | OK | SRPN | 0.0 | 0.0 | 0.0 | 0.0 | S |
| AM070 | AM070 | CO03W043 | HWW | CO | SRPN | 0.0 | 0.0 | 0.0 | 0.0 | S |
| AM071 | AM071 | TX01V5134RC-3 | HRW | TX | SRPN | 0.0 | 0.0 | 0.0 | 0.0 | S |
| AM074 | AM074 | NW03666 | HRW | NE | NRPN | 0.0 | 0.0 | 0.0 | 0.0 | S |
| AM075 | AM075 | MTS0531 | HRW | MT | NRPN | 0.0 | 0.0 | 0.0 | 0.0 | S |
| AM076 | AM076 | Centerfield | HRW | OK | PI644017 | 0.0 | 0.0 | 0.0 | 0.0 | S |
| AM077 | AM077 | OK04525 | HRW | OK | OSU | 0.0 | 0.0 | 0.0 | 0.0 | S |
| AM078 | AM078 | OK03305 | HRW | OK | SRPN | 0.0 | 0.0 | 0.0 | 0.0 | S |
| AM079 | AM079 | MT0552 | HRW | MT | NRPN | 0.0 | 0.0 | 0.0 | 0.0 | S |
| AM080 | AM080 | T154 | HRW | KS | SRPN | 0.0 | 0.0 | 0.0 | 0.0 | S |
| AM083 | AM083 | SD06069 | HRW | SD | NRPN | 0.0 | 0.0 | 0.0 | 0.0 | S |
| AM084 | AM084 | SD05W030 | HWW | SD | NRPN | 0.0 | 0.0 | 0.0 | 0.0 | S |
| AM087 | AM087 | OK05830 | HRW | OK | OSU | 0.0 | 0.0 | 0.0 | 0.0 | S |
| AM088 | AM088 | OK02405 | HRW | OK | RGON | 0.0 | - | 0.0 | 0.0 | S |
| AM092 | AM092 | TX06A001239 | HRW | TX | RGON | 0.0 | 0.0 | 0.0 | 0.0 | S |
| AM097 | AM097 | OK04507 | HRW | OK | RGON | 0.0 | 0.0 | 0.0 | 0.0 | S |
| AM099 | AM099 | KS010143K-11 | HRW | KS | RGON | 0.0 | 0.0 | 0.0 | 0.0 | S |
| AM100 | AM100 | TX05A001334 | HRW | TX | RGON | 0.0 | 0.0 | 0.0 | 0.0 | S |
| AM104 | AM104 | OK05312 | HRW | OK | RGON | 0.0 | 0.0 | 0.0 | 0.0 | S |
| AM107 | AM107 | NW05M6011-6-1 | HWW | NE | RGON | 0.0 | 0.0 | 0.0 | 0.0 | S |
| AM109 | AM109 | TXHT023F7-CS06/607-STA07/40 | HRW | TX | RGON | 0.0 | 0.0 | 0.0 | 0.0 | S |
| AM110 | AM110 | AR97044-10-2 | SRW | AR | UESRWWN | 0.0 | 0.0 | 0.0 | 0.0 | S |
| AM111 | AM111 | P02444A1-23-9 | SRW | IN | UESRWWN | 0.0 | 0.0 | 0.0 | 0.0 | S |
| AM112 | AM112 | VA05W-414 | SRW | VA | UESRWWN | 0.0 | 0.0 | 0.0 | 0.0 | S |
| AM116 | AM116 | NW05M6015-25-4 | HWW | NE | RGON | 0.0 | 0.0 | 0.0 | 0.0 | S |
| AM121 | AM121 | LA01*425 | SRW | IN | UESRWWN | 0.0 | 0.0 | 0.0 | 0.0 | S |
| AM122 | AM122 | KS07HW25 | HWW | KS | RGON | 0.0 | 0.0 | 0.0 | 0.0 | S |
| AM124 | AM124 | KS010379M-2 | HRW | KS | RGON | 0.0 | 0.0 | 0.0 | 0.0 | S |
| AM127 | AM127 | OH02-12678 | SRW | OH | UESRWWN | 0.0 | 0.0 | 0.0 | 0.0 | S |
| AM133 | AM133 | MO040192 | SRW | MO | UESRWWN | 0.0 | 0.0 | 0.0 | 0.0 | S |
| AM135 | AM135 | KS07HW81 | HWW | KS | RGON | 0.0 | 0.0 | 0.0 | 0.0 | S |
| AM136 | AM136 | U07-698-9 | HRW | KS | RGON | 0.0 | 0.0 | 0.0 | 0.0 | S |
| AM137 | AM137 | TX05V5614 | HRW | TX | RGON | 0.0 | 0.0 | 0.0 | 0.0 | S |
| AM143 | AM143 | TX06A001084 | HRW | TX | RGON | 0.0 | 0.0 | 0.0 | 0.0 | S |
| AM144 | AM144 | Bess | SRW | MO | UESRWWN | 0.0 | 0.0 | 0.0 | 0.0 | S |
| AM148 | AM148 | NC04-15533 | SRW | NC | USSRWWN | 0.0 | 0.0 | 0.0 | 0.0 | S |
| AM154 | AM154 | B030543 | SRW | AR | USSRWWN | 0.0 | 0.0 | 0.0 | 0.0 | S |
| AM158 | AM158 | India exp. | SRW | OH | UESRWWN | 0.0 | 0.0 | 0.0 | 0.0 | S |
| AM162 | AM162 | VA05W-78 | SRW | VA | USSRWWN | 0.0 | - | - | 0.0 | S |
| AM163 | AM163 | OK05723W | HRW | OK | OSU | 0.0 | 0.0 | 0.0 | 0.0 | S |
| AM166 | AM166 | D04*5513 | SRW | AR | UESRWWN | 0.0 | 0.0 | 0.0 | 0.0 | S |
| AM167 | AM167 | M04-4566 | SRW | IN | UESRWWN | 0.0 | 0.0 | 0.0 | 0.0 | S |
| AM168 | AM168 | NC03-6228 | SRW | NC | USSRWWN | 0.0 | 0.0 | 0.0 | 0.0 | S |
| AM169 | AM169 | AR96077-7-2 | SRW | AR | USSRWWN | 0.0 | 0.0 | 0.0 | 0.0 | S |
| AM172 | AM172 | OK01420W | HRW | OK | OSU | 0.0 | 0.0 | 0.0 | 0.0 | S |
| AM174 | AM174 | OK06518 | HRW | OK | OSU | 0.0 | 0.0 | 0.0 | 0.0 | S |
| AM175 | AM175 | KY97C-0321-02-01 | SRW | KY | UESRWWN | 0.0 | 0.0 | 0.0 | 0.0 | S |
| AM176 | AM176 | M04-4802 | SRW | IN | UESRWWN | 0.0 | 0.0 | 0.0 | 0.0 | S |
| AM177 | AM177 | AR97124-4-3 | SRW | AR | USSRWWN | 0.0 | 0.0 | 0.0 | 0.0 | S |
| AM179 | AM179 | G61505 | SRW | IN | USSRWWN | 0.0 | 0.0 | 0.0 | 0.0 | S |
| AM182 | AM182 | KY97C-0519-04-07 | SRW | KY | UESRWWN | 0.0 | 0.0 | 0.0 | 0.0 | S |
| AM185 | AM185 | MD01W233-06-1 | SRW | MD | USSRWWN | 0.0 | 0.0 | 0.0 | 0.0 | S |
| AM189 | AM189 | W06-202B | SRW | WI | UESRWWN | 0.0 | 0.0 | 0.0 | 0.0 | S |
| AM190 | AM190 | TAM110 | HRW | TX | PI595757 | 0.0 | 0.0 | 0.0 | 0.0 | S |
| AM191 | AM191 | LA99005UC-31-3-C | SRW | LA | USSRWWN | 0.0 | 0.0 | 0.0 | 0.0 | S |
| AM193 | AM193 | TN801 | SRW | TN | USSRWWN | 0.0 | 0.0 | 0.0 | 0.0 | S |
| AM195 | AM195 | OK05212 | HRW | OK | OSU | 0.0 | 0.0 | 0.0 | 0.0 | S |
| AM196 | AM196 | OK06336 | HRW | OK | OSU | 0.0 | 0.0 | 0.0 | 0.0 | S |
| AM198 | AM198 | AGS 2000 | SRW | GA | USSRWWN | 0.0 | 0.0 | 0.0 | 0.0 | S |
| AM199 | AM199 | LA98214D_14_1_2_B | SRW | LA | USSRWWN | 0.0 | 0.0 | 0.0 | 0.0 | S |

Notes: HRW: hard red winter wheat; HWW: hard white winter wheat; SRW: soft red winter wheat.

AR: Arkansas; CO: Colorado; KS: Kansas; KY: Kentucky; IL: Illinois; IN: Indiana; MA: Maryland; MO: Missouri; NC: North Carolina; NE: Nebraska; OK: Oklahoma; OH: Ohio; SC: South Carolina; SD: South Dakota; TX: Texas; VA: Virgina; WI: Wisconsin.

SRPN: Southern Regional Performance Nursery; NRPN: Northern Regional Performance Nursery; RGON: Regional Germplasm Observation Nursery; USSRWWN: Uniform Southern Soft Red Winter Wheat Nursery; UESRWWN: Uniform Eastern Soft Red Winter Wheat Nursery; OSU: Oklahoma State University.

Table S3 Geographic distribution of Hessian fly resistant accessions screened in this study.

| **Country** | **Number of accessions** | | | | |  | **HR(%)** | **MR(%)** |
| --- | --- | --- | --- | --- | --- | --- | --- | --- |
|  | **Total** | **HR** | **Ratio of HR** | **MR** | **Ratio of MR** |  |  |  |
| United States | 338 | 78 | 23.1 | 44 | 13.0 | 36.1 | 81.3 | 30.6 |
| Iran | 674 | 5 | 0.7 | 36 | 5.3 | 6.1 | 5.2 | 25.0 |
| Azerbaijan | 14 | 2 | 14.3 | 2 | 14.3 | 28.6 | 2.1 | 1.4 |
| Russian Federation | 28 | 2 | 7.1 | 1 | 3.6 | 10.7 | 2.1 | 0.7 |
| Switzerland | 29 | 1 | 3.4 | 2 | 6.9 | 10.3 | 1.0 | 1.4 |
| Croatia | 29 | 1 | 3.4 | 2 | 6.9 | 10.3 | 1.0 | 1.4 |
| Bulgaria | 45 | 1 | 2.2 | 1 | 2.2 | 4.4 | 1.0 | 0.7 |
| France | 36 | 1 | 2.8 | 1 | 2.8 | 5.6 | 1.0 | 0.7 |
| Mexico | 12 | 1 | 8.3 | 1 | 8.3 | 16.7 | 1.0 | 0.7 |
| New Zealand | 11 | 1 | 9.1 | 1 | 9.1 | 18.2 | 1.0 | 0.7 |
| South Korea | 19 | 1 | 5.3 | 1 | 5.3 | 10.5 | 1.0 | 0.7 |
| Former Soviet Union | 14 | 1 | 7.1 | 1 | 7.1 | 14.3 | 1.0 | 0.7 |
| Pakistan | 189 | 1 | 0.5 | 0 | 0.0 | 0.5 | 1.0 | 0.0 |
| Italy | 25 | 0 | 0.0 | 6 | 24.0 | 24.0 | 0.0 | 4.2 |
| Germany | 27 | 0 | 0.0 | 3 | 11.1 | 11.1 | 0.0 | 2.1 |
| Chile | 42 | 0 | 0.0 | 2 | 4.8 | 4.8 | 0.0 | 1.4 |
| United Kingdom | 31 | 0 | 0.0 | 2 | 6.5 | 6.5 | 0.0 | 1.4 |
| Romania | 30 | 0 | 0.0 | 2 | 6.7 | 6.7 | 0.0 | 1.4 |
| Serbia | 32 | 0 | 0.0 | 2 | 6.3 | 6.3 | 0.0 | 1.4 |
| Czech Republic | 25 | 0 | 0.0 | 2 | 8.0 | 8.0 | 0.0 | 1.4 |
| Bosnia and Herzegovina | 21 | 0 | 0.0 | 2 | 9.5 | 9.5 | 0.0 | 1.4 |
| Georgia | 13 | 0 | 0.0 | 2 | 15.4 | 15.4 | 0.0 | 1.4 |
| Portugal | 11 | 0 | 0.0 | 2 | 18.2 | 18.2 | 0.0 | 1.4 |
| Morocco | 6 | 0 | 0.0 | 2 | 33.3 | 33.3 | 0.0 | 1.4 |
| Syria | 4 | 0 | 0.0 | 2 | 50.0 | 50.0 | 0.0 | 1.4 |
| Japan | 47 | 0 | 0.0 | 1 | 2.1 | 2.1 | 0.0 | 0.7 |
| Denmark | 28 | 0 | 0.0 | 1 | 3.6 | 3.6 | 0.0 | 0.7 |
| Kenya | 12 | 0 | 0.0 | 1 | 8.3 | 8.3 | 0.0 | 0.7 |
| Austria | 21 | 0 | 0.0 | 1 | 4.8 | 4.8 | 0.0 | 0.7 |
| Spain | 15 | 0 | 0.0 | 1 | 6.7 | 6.7 | 0.0 | 0.7 |
| Kazakhstan | 10 | 0 | 0.0 | 1 | 10.0 | 10.0 | 0.0 | 0.7 |
| Uzbekistan | 10 | 0 | 0.0 | 1 | 10.0 | 10.0 | 0.0 | 0.7 |
| Peru | 11 | 0 | 0.0 | 1 | 9.1 | 9.1 | 0.0 | 0.7 |
| Uruguay | 9 | 0 | 0.0 | 1 | 11.1 | 11.1 | 0.0 | 0.7 |
| Canada | 8 | 0 | 0.0 | 1 | 12.5 | 12.5 | 0.0 | 0.7 |
| Colombia | 6 | 0 | 0.0 | 1 | 16.7 | 16.7 | 0.0 | 0.7 |
| Ethiopia | 5 | 0 | 0.0 | 1 | 20.0 | 20.0 | 0.0 | 0.7 |
| Iraq | 4 | 0 | 0.0 | 1 | 25.0 | 25.0 | 0.0 | 0.7 |
| Turkmenistan | 4 | 0 | 0.0 | 1 | 25.0 | 25.0 | 0.0 | 0.7 |
| Ecuador | 4 | 0 | 0.0 | 1 | 25.0 | 25.0 | 0.0 | 0.7 |
| Afghanistan | 18 | 0 | 0.0 | 1 | 5.6 | 5.6 | 0.0 | 0.7 |
| Australia | 16 | 0 | 0.0 | 1 | 6.3 | 6.3 | 0.0 | 0.7 |
| India | 7 | 0 | 0.0 | 1 | 14.3 | 14.3 | 0.0 | 0.7 |
| Angola | 5 | 0 | 0.0 | 1 | 20.0 | 20.0 | 0.0 | 0.7 |
| Jordan | 3 | 0 | 0.0 | 1 | 33.3 | 33.3 | 0.0 | 0.7 |
| Oman | 2 | 0 | 0.0 | 1 | 50.0 | 50.0 | 0.0 | 0.7 |
| Europe | 2 | 0 | 0.0 | 1 | 50.0 | 50.0 | 0.0 | 0.7 |
| China | 127 | 0 | 0.0 | 0 | 0.0 | 0.0 | 0.0 | 0.0 |
| Ukraine | 31 | 0 | 0.0 | 0 | 0.0 | 0.0 | 0.0 | 0.0 |
| Belgium | 28 | 0 | 0.0 | 0 | 0.0 | 0.0 | 0.0 | 0.0 |
| Macedonia | 28 | 0 | 0.0 | 0 | 0.0 | 0.0 | 0.0 | 0.0 |
| Hungary | 27 | 0 | 0.0 | 0 | 0.0 | 0.0 | 0.0 | 0.0 |
| Netherlands | 24 | 0 | 0.0 | 0 | 0.0 | 0.0 | 0.0 | 0.0 |
| Sweden | 23 | 0 | 0.0 | 0 | 0.0 | 0.0 | 0.0 | 0.0 |
| Poland | 22 | 0 | 0.0 | 0 | 0.0 | 0.0 | 0.0 | 0.0 |
| Argentina | 16 | 0 | 0.0 | 0 | 0.0 | 0.0 | 0.0 | 0.0 |
| Slovakia | 16 | 0 | 0.0 | 0 | 0.0 | 0.0 | 0.0 | 0.0 |
| Turkey | 15 | 0 | 0.0 | 0 | 0.0 | 0.0 | 0.0 | 0.0 |
| Finland | 11 | 0 | 0.0 | 0 | 0.0 | 0.0 | 0.0 | 0.0 |
| Bhutan | 11 | 0 | 0.0 | 0 | 0.0 | 0.0 | 0.0 | 0.0 |
| Armenia | 10 | 0 | 0.0 | 0 | 0.0 | 0.0 | 0.0 | 0.0 |
| Brazil | 9 | 0 | 0.0 | 0 | 0.0 | 0.0 | 0.0 | 0.0 |
| Israel | 9 | 0 | 0.0 | 0 | 0.0 | 0.0 | 0.0 | 0.0 |
| South Africa | 9 | 0 | 0.0 | 0 | 0.0 | 0.0 | 0.0 | 0.0 |
| Greece | 9 | 0 | 0.0 | 0 | 0.0 | 0.0 | 0.0 | 0.0 |
| Nepal | 8 | 0 | 0.0 | 0 | 0.0 | 0.0 | 0.0 | 0.0 |
| Yemen | 7 | 0 | 0.0 | 0 | 0.0 | 0.0 | 0.0 | 0.0 |
| Guatemala | 6 | 0 | 0.0 | 0 | 0.0 | 0.0 | 0.0 | 0.0 |
| Egypt | 6 | 0 | 0.0 | 0 | 0.0 | 0.0 | 0.0 | 0.0 |
| Montenegro | 6 | 0 | 0.0 | 0 | 0.0 | 0.0 | 0.0 | 0.0 |
| Kyrgyzstan | 5 | 0 | 0.0 | 0 | 0.0 | 0.0 | 0.0 | 0.0 |
| Lebanon | 5 | 0 | 0.0 | 0 | 0.0 | 0.0 | 0.0 | 0.0 |
| Burundi | 4 | 0 | 0.0 | 0 | 0.0 | 0.0 | 0.0 | 0.0 |
| Sudan | 4 | 0 | 0.0 | 0 | 0.0 | 0.0 | 0.0 | 0.0 |
| Tunisia | 4 | 0 | 0.0 | 0 | 0.0 | 0.0 | 0.0 | 0.0 |
| Tajikistan | 4 | 0 | 0.0 | 0 | 0.0 | 0.0 | 0.0 | 0.0 |
| Norway | 4 | 0 | 0.0 | 0 | 0.0 | 0.0 | 0.0 | 0.0 |
| Cyprus | 3 | 0 | 0.0 | 0 | 0.0 | 0.0 | 0.0 | 0.0 |
| Saudi Arabia | 3 | 0 | 0.0 | 0 | 0.0 | 0.0 | 0.0 | 0.0 |
| Algeria | 3 | 0 | 0.0 | 0 | 0.0 | 0.0 | 0.0 | 0.0 |
| Zambia | 3 | 0 | 0.0 | 0 | 0.0 | 0.0 | 0.0 | 0.0 |
| Zimbabwe | 3 | 0 | 0.0 | 0 | 0.0 | 0.0 | 0.0 | 0.0 |
| Belarus | 3 | 0 | 0.0 | 0 | 0.0 | 0.0 | 0.0 | 0.0 |
| Moldova | 3 | 0 | 0.0 | 0 | 0.0 | 0.0 | 0.0 | 0.0 |
| Venezuela | 3 | 0 | 0.0 | 0 | 0.0 | 0.0 | 0.0 | 0.0 |
| Bolivia | 3 | 0 | 0.0 | 0 | 0.0 | 0.0 | 0.0 | 0.0 |
| Eritrea | 2 | 0 | 0.0 | 0 | 0.0 | 0.0 | 0.0 | 0.0 |
| Indonesia | 2 | 0 | 0.0 | 0 | 0.0 | 0.0 | 0.0 | 0.0 |
| Philippines | 2 | 0 | 0.0 | 0 | 0.0 | 0.0 | 0.0 | 0.0 |
| North Korea | 2 | 0 | 0.0 | 0 | 0.0 | 0.0 | 0.0 | 0.0 |
| Estonia | 2 | 0 | 0.0 | 0 | 0.0 | 0.0 | 0.0 | 0.0 |
| Lithuania | 2 | 0 | 0.0 | 0 | 0.0 | 0.0 | 0.0 | 0.0 |
| Former Yugoslavia | 2 | 0 | 0.0 | 0 | 0.0 | 0.0 | 0.0 | 0.0 |
| Mali | 1 | 0 | 0.0 | 0 | 0.0 | 0.0 | 0.0 | 0.0 |
| Albania | 1 | 0 | 0.0 | 0 | 0.0 | 0.0 | 0.0 | 0.0 |
| Chad | 1 | 0 | 0.0 | 0 | 0.0 | 0.0 | 0.0 | 0.0 |
| Libya | 1 | 0 | 0.0 | 0 | 0.0 | 0.0 | 0.0 | 0.0 |
| Malawi | 1 | 0 | 0.0 | 0 | 0.0 | 0.0 | 0.0 | 0.0 |
| Mozambique | 1 | 0 | 0.0 | 0 | 0.0 | 0.0 | 0.0 | 0.0 |
| Nigeria | 1 | 0 | 0.0 | 0 | 0.0 | 0.0 | 0.0 | 0.0 |
| Tanzania | 1 | 0 | 0.0 | 0 | 0.0 | 0.0 | 0.0 | 0.0 |
| Asia | 1 | 0 | 0.0 | 0 | 0.0 | 0.0 | 0.0 | 0.0 |
| Honduras | 1 | 0 | 0.0 | 0 | 0.0 | 0.0 | 0.0 | 0.0 |
| Paraguay | 1 | 0 | 0.0 | 0 | 0.0 | 0.0 | 0.0 | 0.0 |
| Unknown | 4 | 0 | 0.0 | 0 | 0.0 | 0.0 | 0.0 | 0.0 |
| Total | 2496 | 96 | 3.8 | 144 | 5.8 | 9.6 | 100.0 | 100.0 |

Notes: HR, highly resistant accession with > 50% Hessian fly resistance scores; MR, moderately resistant accessions with 1-50% Hessian fly resistance scores; S, completely susceptible accessions with no resistant plant.

HR(%) refers to the ratio of the number of HR accessions in a country to the total number of HR accessions identified in the collection; MR(%) refers to the ratio of the number of MR accessions in a specific country to the total number of MR accessions identified in the collection.

Table S4 Hessian fly resistance scores (%) of the 39 wheat accessions carrying known Hessian fly resistance genes.

| **Genes** | **Accessions** | **Hessian fly resistance score (%)** | **Number of seedlings screened** |
| --- | --- | --- | --- |
| *H1H2* | Dawson | 0.0 | 20 |
| *H3* | Monon | 87.5 | 32 |
| *H3* | Carol | 79.3 | 29 |
| *H3* | Ike | 94.4 | 18 |
| *h4* | Java | 30.0 | 20 |
| *H5* | Magnum | 90.9 | 33 |
| *H5* | Erin | 100.0 | 29 |
| *H6* | Caldwell | 92.3 | 26 |
| *H6* | Flynn | 52.9 | 51 |
| *H6* | Knox 62 | 94.1 | 68 |
| *H7H8* | Seneca | 100.0 | 21 |
| *H9* | Iris | 83.3 | 30 |
| *H10* | Joy | 56.3 | 32 |
| *H11* | Karen | 100.0 | 19 |
| *H12* | Lola | 66.7 | 15 |
| *H13* | Molly | 100.0 | 16 |
| *H14* | 921676A3-5 | 66.7 | 15 |
| *H15* | 81602C5-3-3-8-1 | 84.6 | 13 |
| *H16* | 921682A4-6 | 92.3 | 13 |
| *H16* | Ella | 100.0 | 14 |
| *H17* | 97104A1-6-1-1 | 100.0 | 25 |
| *H17* | 921680d1-7 | 100.0 | 10 |
| *H18* | 97104A1-6-1-3 | 100.0 | 25 |
| *H18* | Redland | 88.9 | 9 |
| *H19* | 84702B14-1-3-4-3 | 88.2 | 17 |
| *H20* | Jori | 90.2 | 51 |
| *H21* | Hamlet | 43.8 | 32 |
| *H22* | KS85WGRC01 | 100.0 | 27 |
| *H23* | KS89WGRC03 | 43.5 | 23 |
| *H24* | KS89WGRC06 | 37.9 | 29 |
| *H25* | KS92WGRC20 | 88.0 | 25 |
| *H26* | KS93WGRC26 | 100.0 | 24 |
| *H28* | PI59190 | 100.0 | 11 |
| *H29* | Sincape90 | 100.0 | 12 |
| *H31* | 921696-H31 | 33.3 | 12 |
| *H32* | Synthetic | 97.0 | 33 |
| *H34* | L118 | 100.0 | 18 |
| *H35H36* | SD06165 | 55.6 | 18 |
| *Hdic* | KS99WGRC42 | 100.0 | 21 |
